# The Bacterial Effector HopX1 Targets JAZ Transcriptional Repressors to Activate Jasmonate Signaling and Promote Infection in *Arabidopsis*

**DOI:** 10.1371/journal.pbio.1001792

**Published:** 2014-02-18

**Authors:** Selena Gimenez-Ibanez, Marta Boter, Gemma Fernández-Barbero, Andrea Chini, John P. Rathjen, Roberto Solano

**Affiliations:** 1 Departamento de Genética Molecular de Plantas, Centro Nacional de Biotecnología-Consejo Superior de Investigaciones Científicas, Madrid, Spain; 2 Research School of Biology, Australian National University, Canberra, Australia; The University of North Carolina at Chapel Hill, United States of America

## Abstract

Pathogenicity of *Pseudomonas syringae* is dependent on a type III secretion system, which secretes a suite of virulence effector proteins into the host cytoplasm, and the production of a number of toxins such as coronatine (COR), which is a mimic of the plant hormone jasmonate-isoleuce (JA-Ile). Inside the plant cell, effectors target host molecules to subvert the host cell physiology and disrupt defenses. However, despite the fact that elucidating effector action is essential to understanding bacterial pathogenesis, the molecular function and host targets of the vast majority of effectors remain largely unknown. Here, we found that effector HopX1 from *Pseudomonas syringae* pv. *tabaci* (*Pta*) 11528, a strain that does not produce COR, interacts with and promotes the degradation of JAZ proteins, a key family of JA-repressors. We show that *hopX1* encodes a cysteine protease, activity that is required for degradation of JAZs by HopX1. HopX1 associates with JAZ proteins through its central ZIM domain and degradation occurs in a COI1-independent manner. Moreover, ectopic expression of HopX1 in *Arabidopsis* induces the expression of JA-dependent genes, represses salicylic acid (SA)-induced markers, and complements the growth of a COR-deficient *P. syringae* pv. *tomato* (*Pto*) DC3000 strain during natural bacterial infections. Furthermore, HopX1 promoted susceptibility when delivered by the natural type III secretion system, to a similar extent as the addition of COR, and this effect was dependent on its catalytic activity. Altogether, our results indicate that JAZ proteins are direct targets of bacterial effectors to promote activation of JA-induced defenses and susceptibility in *Arabidopsis*. HopX1 illustrates a paradigm of an alternative evolutionary solution to COR with similar physiological outcome.

## Abstract 2

A bacterial effector protein, HopX1, targets host plant JAZ transcriptional repressors for degradation to activate the jasmonate pathway, thereby promoting bacterial pathogenesis by suppressing host defense responses.

## Author Summary

Bacterial plant pathogens secrete toxins and inject effector proteins into the host cells to promote infection, and the identification of the individual functions of these molecules is essential to understand the infective process. Remarkably, some Pseudomonas strains have evolved a sophisticated strategy for manipulating hormonal balance by producing the toxin coronatine (COR), which mimics the plant hormone jasmonate-isoleucine (JA-Ile). The JA-Ile pathway plays a key role in plant immunity by activating defenses against fungal pathogens, while promoting bacterial growth by inhibiting the salicylic acid (SA)-dependent defenses required for *Pseudomonas* resistance. Here, we report that the effector HopX1 from a *Pseudomonas syringae* strain that does not produce COR exploits an alternative evolutionary strategy to activate the JA-Ile pathway. We show that HopX1 encodes a cysteine protease that interacts with and promotes the degradation of key JA pathway repressors, the JAZ proteins. Correspondingly, ectopically expressing HopX1 in the model plant *Arabidopsis* induces the expression of JA-dependent genes, and natural infection with *Pseudomonas* producing HopX1 promotes bacterial growth in a similar fashion to COR. Our results highlight a novel example by which a bacterial effector directly manipulates core regulators of hormone signaling to facilitate infection.

## Introduction

*Pseudomonas syringae* is a widespread bacterial pathogen that causes disease on a broad range of economically important plant species. In order to infect, *P. syringae* produces a number of toxins and uses a type III secretion system (TTSS) to deliver effector proteins into eukaryotic cells [1],[2]. This mechanism is essential for successful infection by both plant- and animal-associated bacteria as bacterial mutants deficient in the TTSS are no longer pathogenic [3]. Effectors contribute collectively to pathogenesis inside the host cell by targeting host molecules and defeating plant defenses, which are based on two tiers of recognition by the innate immune system [4]. The first branch is triggered by the recognition of highly conserved microbe-associated molecular patterns (MAMPs) by host cell transmembrane proteins that function as pattern recognition receptors (PRRs), which in turn, activate MAMP-triggered immunity (MTI) [4]. The second branch recognizes type III effectors inside the plant cell via nucleotide-binding site-leucine-rich repeat (NB-LRR) resistance (R) proteins [4]. This leads to activation of effector-triggered immunity (ETI), and is characteristically associated with programmed cell death known as the hypersensitive response (HR). Accumulating evidence suggests that a primary function of microbial effectors is suppression of both MTI and ETI to avoid pathogen recognition during the infection process [5]. However, despite the fact that elucidating effector action is essential to understanding bacterial pathogenesis, the molecular function and host targets of the vast majority of effectors remain largely unknown.

Plant immunity relies on a complex network of small-molecule hormone signaling pathways [6]. Classically, salicylic acid (SA) signaling mediates resistance against biotrophic and hemi-biotrophic microbes such as *P. syringae*, whereas a combination of jasmonic acid (JA) and ethylene (ET) pathways activates resistance against necrotrophs such as the fungal pathogen *Botrytis cinerea* [6]. SA and JA/ET defense pathways generally antagonize each other and thus, elevated resistance against biotrophs is often correlated with increased susceptibility to necrotrophs, and vice versa [7]. The collective contribution of these two hormones during plant-pathogen interactions is crucial to the success of the interaction. Remarkably, some *P. syringae* strains have evolved a sophisticated strategy for manipulating hormonal homeostasis by producing coronatine (COR), a mimic of the bioactive jasmonate hormone, JA-isoleucine (JA-Ile) [8]. COR contributes to disease symptomatology by inducing chlorotic lesions [9]–[11], facilitates entry of the bacteria into the plant host by stimulating the opening of stomata [12],[13], and promotes bacterial growth by inhibiting SA-dependent defenses required for *P. syringae* resistance, because of its activation of the antagonistic JA pathway [14],[15]. COR, as the JA-Ile phytohormone, is perceived through a receptor complex formed by the F-box protein CORONATINE-INSENSITIVE 1 (COI1) and JASMONATE ZIM DOMAIN (JAZ) proteins [16]–[18]. COI1 is the F-box component of an SCF-(Skip-cullin-F-box)-type E3 ubiquitin ligase required for all JA-dependent responses tested so far [8],[19]–[22]. JAZ co-receptors are COI1 substrates that negatively regulate the JA-signaling pathway by directly interacting with and repressing transcription factors (TFs) that control JA-regulated genes [16]–[18],[23],[24]. Repression of TFs by JAZ is mediated by a general co-repressor machinery involving TOPLESS (TPL) and TPL-related proteins that interact with JAZ repressors through the adaptor protein NINJA [25]. The JAZ family of JA-repressors consists of 12 members in *Arabidopsis* that have emerged as central modulators of JA signaling [17],[18],[26]. Under stress conditions, COR or JA-Ile promotes the formation of JAZ-COI1 complexes, triggering JAZ degradation via the 26S proteasome [16]–[18]. This leads to de-repression of the TFs that initiate the transcription of JA-dependent genes, and repression of SA-dependent defenses against the bacteria. Thus, COR acts as a potent virulence factor in plants by triggering the degradation of JAZs. Acquisition of COR by bacterial pathogens has been of tremendous adaptive importance during host-pathogen evolution because it has allowed bacteria to manipulate the host hormonal network to promote susceptibility.

COR is produced by several bacterial strains distributed throughout the *P. syringae* phylogeny, but is particularly common among *P. syringae* pv. *tomato* strains such as DC3000 (*Pto* DC3000) [27],[28]. Interestingly, strains such as *P. syringae* pv. *tabac*i (*Pta*) 11528 that do not produce COR can still open stomata, suggesting that other virulence factors, probably type-III effectors, are used to activate the JA pathway instead of COR [12],[29],[30]. This is supported by several studies suggesting that COR and effectors act synergistically to induce JA responses [31]–[35]. Indeed, several effectors have been shown to modulate the expression of JA-inducible genes [33]. Moreover, gene expression profiles indicate that several JA-regulated genes are still induced in *coi1* mutants upon *P. syringae* infection, indicating that JA responses are activated downstream or independently of COI1 [34]. Therefore, bacterial effectors should target other components of the JA pathway downstream of COI1 to activate JA responses, the best candidates being JAZ repressors. Recently, Mukhtar and colleagues developed a large-scale map of physical interactions between proteins from the reference plant *Arabidopsis thaliana* and effector proteins from *P. syringae* and the obligate biotrophic oomycete *Hyaloperonospora arabidopsidis* (*Hpa*) [36]. The experiment yielded a map of 6,200 interactions, and showed that pathogens from different kingdoms deploy independently evolved virulence effectors that interact with a limited set of highly connected cellular hubs to facilitate their diverse life-cycle strategies. Strikingly, two out of the five most significantly targeted plant hub proteins by effectors (namely RESPONSE TO LOW SULPHUR [LSU1, AT3G49580] and an unknown kinesin light-chain related protein [AT3G27960]), physically interact with several JAZ proteins [36]. Thus, it is plausible that pathogens may attempt to manipulate the JA pathway directly through JAZ proteins.

In previous studies, we generated a draft genome sequence of *Pta* 11528 and used a functional screen to infer its repertoire of T3SS effectors [30]. This led to the identification of *Pta* 11528 proteins with homology to previously described effectors. Since *Pta* 11528 does not produce COR, we hypothesized that it might have followed an alternative evolutionary strategy to activate the JA pathway by developing effector proteins that target JA signaling components. As noted above, we hypothesized that JAZ repressors would be the best candidate targets. Therefore, to study whether any such effector proteins target JAZ repressors, we developed a screen to analyze the stability of JAZ proteins in the presence of each *Pta* 11528 effector. We found that the *Pta* 11528 effector HopX1 encodes a cysteine protease that interacts with and degrades JAZ proteins in a COI1-independent manner. Ectopic expression of HopX1 in *Arabidopsis* induced the expression of JA-dependent genes, compromised the induction of SA-marker gene *PR1* upon SA treatment, and complemented the growth of a COR-deficient *Pto* DC3000 strain during natural bacterial infections. Moreover, *Pto* DC3000 *COR−* growth increased by about one log (colony forming units [cfu]/cm^2^) when naturally expressing *hopX1*, but not its catalytic mutant version, indicating that HopX1 can effectively promote susceptibility when delivered by the natural TTSS. This increase in susceptibility was similar to the effect of supplementing bacteria with 2 µM of COR and independent of COI1. Altogether, the results indicate that similar to COR, HopX1 acts within the plant cell to promote activation of jasmonate-induced defenses and bacterial disease bypassing the need of JA-Ile perception.

## Results

### HopX1 Compromises the Accumulation of JAZ Proteins

It has been suggested that *P. syringae* pv. *tabaci* activates JA responses such as stomatal aperture without producing COR [12],[29],[30], which suggests that effectors from this strain could provide the same function by targeting components of the JA pathway. Whereas stomatal closure is part of a plant innate immune response triggered upon pathogen perception to restrict bacterial invasion, plant pathogenic bacteria have evolved specific virulence factors such as COR to promote stomata opening in order to circumvent such innate immune responses [12],[13]. To investigate whether live *Pta* 11528 bacteria can re-open plant stomata without producing COR, we first incubated host *Nicotiana benthamiana* leaves with virulent *Pta* 11528 bacteria. In this experiment, *Pto* DC3000 was used as a positive control, and a non-pathogenic TTSS defective strain *Pta* 11528 *hrpV−* unable to secrete effector proteins into the plant cell served as a negative control [37]. Incubation of *N. benthamiana* leaves with *Pta* 11528 *hrpV−* for 5 hours induced stomatal closure as described previously (Figure S1) [12]. In contrast, virulent *Pta* 11528 or *Pto* DC3000 bacteria maintained stomatal apertures similar to mock treatments. This indicates that effector proteins from *Pta* 11528 might play a role in re-opening of stomata as COR does, probably through action on the JA-signaling pathway.

**Figure 1:**
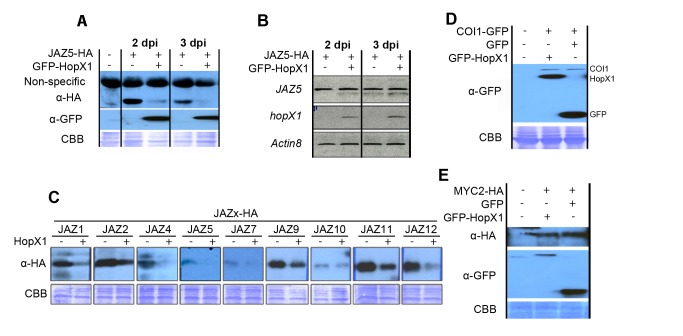
HopX1 compromises the accumulation of JAZ proteins. (A) HopX1 compromises the accumulation of JAZ5. Immunoblots showing JAZ5-HA accumulation in the presence of GFP-HopX1 when co-expressed transiently in *N. benthamiana*. Proteins were detected with anti-HA and anti-GFP antisera respectively. A non-specific band is shown as an internal loading control. CBB, Coomassie brilliant blue staining. This experiment was repeated four times with similar results. (B) HopX1 does not affect JAZ5 expression levels. RT-PCRs showing transgenic *JAZ5* mRNA in *N. benthamiana* leaves transiently co-expressing JAZ5 with an EV control or GFP-HopX1. *Actin8* was used as an amplification control. dpi, days post infiltration. This experiment was repeated twice with similar results. (C) HopX1 activity is not restricted to JAZ5, but targets all detectable JAZs. Immunoblots showing the accumulation of eight JAZ-HA proteins in the presence of an EV control or GFP-HopX1 when co-expressed transiently in *N. benthamiana*. This experiment was repeated twice with similar results. (D) HopX1 does not alter COI1 proteins levels. Immunoblots showing COI1-GFP accumulation in the presence of GFP-HopX1 when co-expressed transiently in *N. benthamiana*. This experiment was repeated twice with similar results. (**E**) HopX1 does not alter MYC2 proteins levels. Immunoblots showing MYC2-HA accumulation in the presence of GFP-HopX1 when co-expressed transiently in *N. benthamiana*. This experiment was repeated twice with similar results.

To identify *Pta* 11528 effector proteins that could target JAZs, we analyzed a *Pta* 11528 effector library constructed in a binary vector within the T-DNA region, in which individual genes are expressed from the *35S* promoter as genetic fusions to three C-terminal hemagglutinin (HA) epitope tags (Table S1). We transiently co-expressed *JAZ5-HA* with individual effector genes from this library or an empty vector (*EV*) control in *N. benthamiana* by agroinfiltration. Using this approach, we identified HopX1 as a *Pta* 11528 effector capable of compromising JAZ5 accumulation (Figure S2). To confirm this result and to exclude a potential effect of the HA tag, we developed an independent form of HopX1 with an N-terminal green fluorescent protein (GFP) fusion. We then transiently co-expressed an *EV* construct or *GFP-hopX1* under the control of the *35S* promoter with *35S:JAZ5-HA* in *N. benthamiana* leaf tissue. Western blot analysis showed that GFP-HopX1 accumulated in *N. benthamiana* (Figure 1A). Similar to previous results, JAZ5-HA protein was detectable when co-expressed with the EV control but not (or only weakly) with GFP-HopX1, despite the fact that *GFP-hopX1* co-expression did not affect *JAZ5-HA* mRNA expression levels (Figure 1B). These results indicate that HopX1 compromised the accumulation of JAZ5 protein when transiently co-expressed in *N. benthamiana* without affecting gene expression levels.

The *Arabidopsis* JAZ family contains 12 members grouped into four major phylogenetic clades (Figure S3) [17],[18],[26]. To test if HopX1 could compromise the accumulation of JAZ proteins other than JAZ5, we transiently co-expressed the 12 *JAZ*-HA genes individually with *GFP-hopX1* or an *EV* control in *N. benthamiana*, and analyzed JAZ accumulation by Western blotting. We successfully detected eight out of 12 JAZ proteins when co-infiltrated with the EV control (Figure 1C). Interestingly, HopX1 compromised the accumulation of all eight JAZ proteins detected, indicating that HopX1 activity is not restricted to JAZ5 but targets the whole JAZ family (Figure 1C). We also analyzed the effect of HopX1 on the stability of additional JA related proteins such as the JA receptor COI1 and the downstream TF MYC2, which is a direct target of JAZ repressors [17]. HopX1 did not alter COI1 or MYC2 protein levels compared to the EV control (Figure 1D and 1E). Altogether, these results indicate that HopX1 compromises the accumulation of the JAZ family of JA-repressors in a specific manner.

### *HopX*1 Encodes a Cysteine Protease and This Activity Is Required for HopX1-Mediated JAZs Degradation

HopX1 family members from different *P. syringae* strains are modular proteins that contain a putative cysteine-based catalytic triad and a novel conserved N-terminal domain [38]. The putative catalytic triad is required for effector function and consists of cysteine (C), histidine (H), and aspartic acid (D) residues conserved with cysteine proteases [38]. In order to determine whether these domains are conserved in HopX1 from *Pta* 11528, we performed a PSI-BLAST search with the HopX1*_Pta_*_ 11528_ protein sequence. BLAST analysis revealed a near identical match with HopX1 from *P. syringae* pv. *phaseolicola* bacterial strains such as race 4 (99% identity), but showed significantly less homology with the respective *Pto* DC3000 homolog (72% identity) (Figure S4A). The putative catalytic triad residues were strictly conserved in HopX1*_Pta_*_ 11528_, as was the novel N-terminal domain of unknown function typical of this family of effectors (Figure S4A). This conservation suggests that the domains may play an important role in the activity of this effector inside the plant cell, and that HopX1*_Pta_*_ 11528_ might have cysteine protease activity.

**Figure 2:**
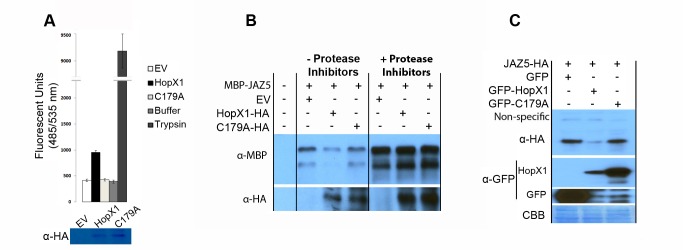
*HopX1* encodes a putative cysteine protease and this activity is required for HopX1-mediated degradation of JAZs. (A) HopX1 has protease activity *in vitro* on the general substrate casein when immunoprecipitated from transgenic *Arabidopsis* plants expressing the transgene. HopX1 or HopX1^C179A^-HA purified under non-denaturing conditions from transgenic *Arabidopsis* plants incubated with fluorescein isothiocyanate (FITC)-labeled casein. Trypsin was used as a positive control. As a negative control, we included wild-type Col-0 plants (EV) subjected to the same immunoprecipitation procedure as for the transgenic plants. Immunoblots showing HopX1-HA and HopX1^C179A^-HA effector inputs are also shown. The results are representative of three independent experiments performed with three independent immunoprecipitations of HopX1-HA and HopX1^C179A^-HA from transgenic *Arabidopisis* plants. (B) HopX1 has protease activity on JAZs when immunoprecipitated from transgenic *Arabidopsis* plants expressing the transgene. The immunoblot shows MBP-JAZ5 accumulation after incubation with immunoprecipitated HopX1-HA or HopX1^C179A^-HA from transgenic *Arabidopsis* plants in the presence or not of protease inhibitors. As a negative control, we included wild-type Col-0 plants (EV) subject to the same immunoprecipitation procedure as for the transgenic plants. The results are representative of three independent experiments performed as in (A). (C) Degradation of JAZ5 by HopX1 requires the cysteine-based catalytic triad of a putative protease *in vivo*. The immunoblots show JAZ5-HA accumulation in the presence of GFP-HopX1, GFP-HopX1^C179A^ or GFP alone when co-expressed transiently in *N. benthamiana*. This experiment was repeated three times with similar results.

To determine if HopX1 has cysteine protease activity *in vitro*, we used a kit designed for the detection of protease activity (serine, aspartic, cysteine, and metalloproteinases) using fluorometry based on the hydrolysis of a labeled casein general substrate [39]. As previously described by Nimchuck and colleagues [38], we did not detect any protease activity when purified recombinant HopX1 protein fused to maltose binding protein (MBP) was incubated with the casein-labeled substrate *in vitro*, indicating that this recombinant protein may be inactive or that it might lack a co-factor (Figure S4B). However, we detected significant protease activity when the casein substrate was incubated with HopX1-HA immunopurified directly from stable transgenic *Arabidopsis* plants expressing the *hopX1* gene from a *dexamethasone*-inducible promoter (*DEX*) (Figure 2A), suggesting that HopX1 expressed *in planta* has protease activity. To test whether this activity of HopX1 required its conserved cysteine protease catalytic triad, we substituted the conserved Cys-179 residue within this domain for an alanine residue to generate the HopX1^C179A^ mutant. HopX1^C179A^-HA inmunoprecipitated from transgenic *Arabidopsis* plants did not show any proteolytic activity compared to negative controls. The trypsin enzyme used as a positive control in these experiments showed much higher activity on the casein substrate than HopX1 (Figure 2A). These data indicate that HopX1 has protease activity, but seems to operate suboptimally on a general substrate *in vitro*. To test whether HopX1 may have evolved specific substrate selectivity, we incubated inmunoprecipitated HopX1 and HopX1^C179A^ from *Arabidopsis* with recombinant MBP-JAZ5 expressed and purified from *Escherichia coli* cells with or without protease inhibitors. The amount of MBP-JAZ5 diminished significantly when incubated with HopX1 but not with HopX1^C179A^ or buffer in the absence of protease inhibitors, but not in its presence (Figure 2B). Thus, HopX1, but not HopX1^C179A^, is capable of inducing JAZ5 degradation *in vitro* suggesting that the effector indeed acts as a protease on the JAZ5 substrate.

To confirm that degradation of JAZ proteins by HopX1 requires its putative cysteine protease activity *in vivo*, we next tested the effect of the C179A mutation on JAZ5-HA accumulation when the proteins were co-expressed transiently in *N. benthamiana* leaves (Figure 2C). JAZ5-HA was detectable in the presence of HopX1^C179A^, but not HopX1, indicating that this catalytic residue is indeed critical for the effect of HopX1 on JAZ5 levels (Figure 2C). Similarly, only wild-type HopX1, but not HopX1^C179A^, compromised the accumulation of additional JAZs such as JAZ1, JAZ2, JAZ9, and JAZ10 when transiently co-expressed in *N. benthamiana* leaves. However, HopX1 did not alter MYC2 protein levels (Figure S4C). To exclude that JAZ5 degradation is a general property of cysteine proteases, we analyzed the effects of previously described cysteine proteases such as HopC1 [40] and HopN1 [41] or an independent HopAD1 effector on the stability of JAZ5 when coexpressed transiently in *N. benthamiana*. As expected, JAZ5-HA accumulation was compromised in the presence of HopX1, but not when co-expressed with the EV control or with HopC1 or HopN1 proteins (Figure S5). The HopAD1 effector could not be detected in this assay. Taken together, these data suggest that degradation of JAZ proteins by HopX1 is specific and depends on a cysteine protease enzymatic activity that requires a conserved cysteine within the proposed catalytic triad.

### HopX1 Associates with and Degrades JAZs in a COI1-Independent Manner

**Figure 3:**
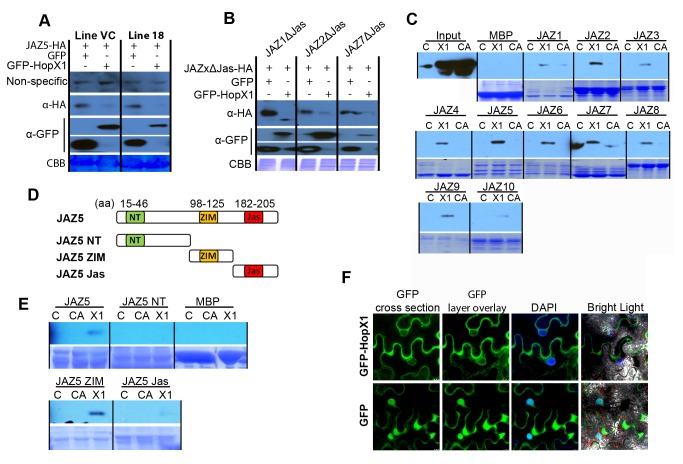
HopX1 interacts with and degrades JAZ proteins in a COI1-independent manner. (A) HopX1 compromises the accumulation of JAZ5 in *N. tabacum* plants silenced for the *NtCOI1* gene. Immunoblots showing JAZ5-HA accumulation in the presence of GFP-HopX1 or GFP alone, when co-expressed transiently in *N. tabacum* plants silenced for the *NtCOI1* gene (line 18) or EV-transformed (line VC). CBB, Coomassie brilliant blue staining. This experiment was repeated three times with similar results. (B) HopX1 triggers the degradation of JAZΔJas proteins in a *COI1*-independent manner. *N. benthamiana* plants were transiently co-transformed with *GFP-hopX1* or *GFP* alone, and the dominant-negative JAZ variants *JAZ1*Δ*Jas-HA*, *JAZ2*Δ*Jas-HA*, or *JAZ7*Δ*Jas-HA* proteins as indicated. Protein stability was analyzed by immunoblot. This experiment was repeated twice times with similar results. (C) HopX1 interacts with JAZ repressors in PD assays. Immunoblots with anti-HA antibody of HopX1-HA or HopX1^C179A^-HA recovered after PD experiments using crude protein extracts from *DEX*:*hopX1-HA* (X1), *DEX*:*hopX1* ^C179A^-*HA* (CA), or Col-0 (C) *Arabidopsis* plants, and resin-bound recombinant MBP or MBP-fused JAZ proteins (top). Input lanes show the level of expression of recombinant HopX1 proteins in transgenic and control plants. CBB staining shows the amount of recombinant JAZ-MBP or MBP proteins used in the resin (bottom). The results are representative of five independent experiments. (D) Schematic representation of the JAZ5 protein and its conserved domains. The NT, the ZIM, and the Jas domains are depicted and the corresponding JAZ5 fragments are represented. (E) HopX1 interacts with JAZ proteins through their conserved ZIM domains in PD assays. Immunoblot (anti-HA antibody) of HopX1-HA and HopX1^C179A^-HA recovered from PD reactions (using extracts of *DEX:hopX1-HA* [X1], *DEX:hopX1* ^C179A^-*HA* [CA], or Col-0 [C] *Arabidopsis* plants) using MBP or MBP-fused JAZ5, JAZ5_1–91_ (JAZ5 NT), JAZ5_92–163_ (JAZ5 ZIM), or JAZ5_164–274_ (JAZ5 Jas) derivatives (top). The lower panels show the CBB staining of the input quantity of recombinant MBP proteins used on the column. The results are representative of three independent experiments. (F) Subcellular localization of HopX1 in plant cells. Confocal microscopy localization of transiently expressed GFP-HopX1 or GFP alone in *N. benthamiana* leaves 48 hours post-infiltration (green). Nuclei were stained with DAPI (blue). This experiment was repeated three times with similar results.

Increased JA-Ile levels promote binding of JAZs to SCF^COI1^ and subsequent degradation of JAZ repressors via the ubiquitin/26S proteasome pathway [16]–[18]. Therefore, degradation of JAZs by HopX1 might be direct (through its protease activity) or an indirect effect mediated by JA-Ile synthesis and COI1. To investigate if JAZ degradation by HopX1 is direct or indirect, we first analyzed whether HopX1-induced degradation of JAZ5 was dependent on the 26S proteasome pathway by using the proteasomal inhibitor MG132. In HopX1 coexpression experiments in *N. benthamiana*, JAZ5 was not detected in the presence of MG132, indicating that degradation does not require the proteasome (Figure S6). We next checked whether HopX1-induced degradation of JAZ proteins occurred in a COI1-dependent or independent manner. To test this, we first used a stable transgenic *N. tabacum* line silenced for expression of the *NtCOI1* gene [42]. Reverse transcription (RT)-PCR analysis confirmed that control *N. tabacum* plants (Line VC, transformed with *EV*) accumulated *NtCOI1* mRNA, whereas *NtCOI1* transcripts were undetectable in *N. tabacum* plants silenced for the *NtCOI1* gene (Line L18) (Figure S7A). Interestingly, *NtCOI1*-silenced *N. tabacum* plants produced few seeds, a phenotype reminiscent of infertile *Arabidopsis coi1-1* plants (Figure S7B) [19]. We next analyzed the ability of HopX1 to trigger JAZ5 degradation in both *EV*- and *NtCOI1*-silenced plants when transiently co-expressed in *N. tabacum*, a species that also allows facile transient gene expression assays [43]. Strikingly, GFP-HopX1 compromised the accumulation of JAZ5 in both *EV*- and *NtCOI1*-silenced plants to the same extent (Figure 3A). This suggests that HopX1 triggers the degradation of JAZ proteins in a COI1-independent manner.

JAZ proteins are characterized by three sequence motifs, namely a relatively conserved N-terminal (NT) motif and the two highly conserved ZIM (central) and Jas (C-terminal) domains (Figure 3D) [17],[18],[23],[26],[44]. The ZIM domain mediates homo- and heteromeric interactions between *Arabidopsis* JAZ proteins [21],[45] and interacts with the general adaptor protein NINJA [25], whereas the C-terminal Jas domain is responsible for the interaction with COI1 and the TFs [21],[24],[46]. Dominant-negative JAZ variants lack part of the C-terminal Jas domain [16],[17],[22]. Consistently, these truncated JAZ forms (JAZΔJas) are resistant to COI1-dependent degradation after jasmonate treatment, and plants overexpressing them are JA-insensitive [17],[18]. To confirm whether degradation of JAZ proteins by HopX1 is COI1-independent, we analyzed the effect of HopX1 on three JAZΔJas proteins, namely JAZ1ΔJas, JAZ2ΔJas, and JAZ7ΔJas. We detected *JAZ1*Δ*Jas*, *JAZ2*Δ*Jas*, and *JAZ7*Δ*Jas* proteins when co-expressed individually with the *EV* control in *N. benthamiana* leaf tissue (Figure 3B). However, none of these JAZΔJas protein forms accumulated when co-expressed with GFP-HopX1 (Figure 3B), confirming that HopX1 triggers the degradation of JAZ proteins in a COI1-independent manner.

All of our previous results imply that HopX1 targets JAZ proteins directly. Therefore, we next examined the ability of HopX1 and HopX1^C179A^ to interact with all 12 *Arabidopsis* JAZ proteins in pull-down (PD) experiments. To do this, we used recombinant JAZ proteins fused to MBP and cell extracts of either wild-type plants (as negative controls), or transgenic plants expressing HopX1-HA or HopX1^C179A^-HA from the *DEX* inducible promoter. These experiments included protease inhibitors so that degradation of JAZs would not diminish a potential interaction with HopX1. As shown in Figure 3C, all full-length MBP–JAZ proteins tested interacted with wild-type HopX1-HA, but not with the mutant HopX1^C179A^-HA form. Similarly, MBP-HopX1 purified from *E. coli* cells co-immunoprecipitated with JAZ5-GFP when the recombinant effector protein was incubated with *N. benthamiana* plants extracts transiently expressing the JAZ5 transgene (Figure S8). We could also detect weak interaction with MBP-HopX1^C179A^ but to a lesser extent than MBP-HopX1. Overall, this indicates that HopX1 interacts with JAZ proteins.

To further determine the requirements for HopX1 interaction with JAZ proteins, we expressed the JAZ deletion mutants JAZ5_1–91_ (NT domain), JAZ5_92–163_ (ZIM domain), and JAZ5_164–274_ (Jas domain) fused to MBP in *E. coli* and purified these fragments for PD analysis with cell extracts of transgenic plants expressing HopX1-HA (Figure 3D). As shown in Figure 3E the N-terminal fragment alone, or the C-terminus containing the Jas domain, did not interact with HopX1. However, the JAZ5_92–163_ derivative containing the ZIM domain was sufficient for interaction with HopX1. Notably, the mutant version HopX1^C179A^ could not be pulled down with any fragment of JAZ5, as we observed previously with full-length JAZ proteins (Figure 3E). The results indicate that HopX1 interacts directly with JAZ proteins though the central ZIM domain, whereas the other conserved domains seem to be dispensable. The data support our previous results showing that HopX1 triggers the degradation of all JAZ proteins, as the ZIM domain is present in all members of the JAZ family of repressors.

JAZ proteins are translated in the cytoplasm and localized predominantly in the nucleus [17],[18]. We next examined in which subcellular compartment the degradation of JAZs proteins by HopX1 takes place. To test this, we expressed GFP or GFP-HopX1 in *N. benthamiana* leaves and analyzed its subcellular localization using confocal microscopy. As reported previously [38], the fluorescent signal corresponding to GFP-HopX1 accumulated mainly in the cytoplasm 48 hours post inoculation (HPI) (Figure 3F). We could also detect GFP-HopX1 fusion protein within the nucleus in cross-middle nuclear sections using confocal microscopy analysis (Figures 3F and S9A), indicating that a pool of the effector enters the nucleus. To confirm this result, we further performed separation of nuclear extracts from the cytoplasmic fraction of *N. benthamiana* leaves transiently expressing GFP alone or GFP-HopX1 fusion protein. Crude fractions enriched for nuclei contained detectable levels of full length GFP-HopX1 although to a lesser extent than cytoplasmic fractions, confirming the microscopy results (Figure S9B). These results indicate that HopX1 could potentially degrade JAZ proteins both in the cytoplasm and nucleus.

### HopX1 Activates JA-Dependent Gene Expression in *Arabidopsis*

**Figure 4:**
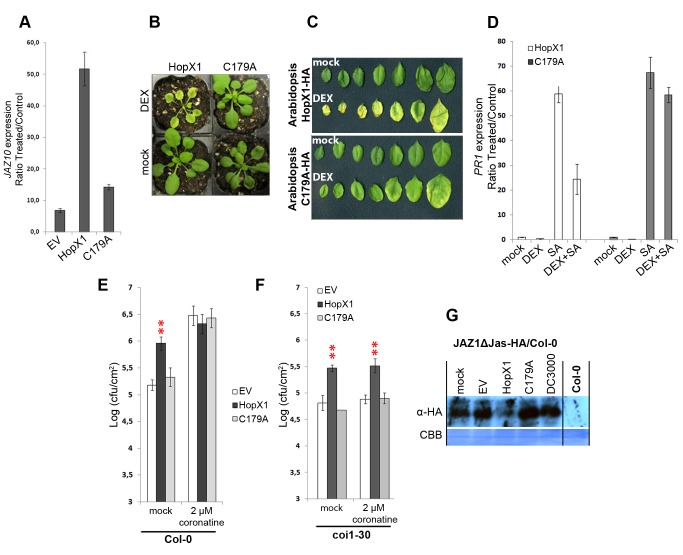
HopX1 activates JA-dependent gene expression in *Arabidopsis*. (**A**) Quantitative RT-PCR analysis of *JAZ10* expression in Col-0 (EV) and stable transgenic *Arabidopsis* Aa–0 lines expressing the *hopX1* or *hopX1* ^C179A^ genes 36 hours after treatment with DEX or a mock solution. The measurements (three technical replicates) represent the expression level between mock (control) and DEX-treated plants relative to each *Arabidopsis* background. All samples were normalized against the housekeeping gene *AtACT8*. Error bars represent standard deviation (SD). The results are representative of four independent experiments. The results are representative of four independent experiments. (**B**) Phenotypes of stable transgenic *Arabidopsis* lines expressing the *hopX1* or *hopX1* ^C179A^ genes under the control of the *DEX* promoter. Pictures were taken six days after mock or DEX treatment. (**C**) Chlorotic phenotypes of stable transgenic *Arabidopsis* lines expressing the *hopX1* or *hopX1* ^C179A^ genes under the control of the *DEX* inducible promoter. Pictures were taken nine days after mock or DEX treatment. (**D**) Quantitative RT-PCR analysis of *PR1* expression in stable transgenic *Arabidopsis* lines expressing the *hopX1* or *hopX1* ^C179A^ genes DEX-induced for 24 hours followed by a treatment with 1 mM SA or a mock solution for an additional 24 hours. The measurements (three technical replicates) represent the ratio of expression levels between control (non-DEX and non-SA treated plants) and treated plants in each *Arabidopsis* background. All samples were normalized against the housekeeping gene *AtACT8*. Error bars represent standard deviation (SD). The results are representative of two independent experiments. This experiment was repeated twice with similar results. (**E**) HopX1 mimics COR-induced susceptibility. Growth of a COR-deficient *Pto* DC3000 strain expressing *hopX1*, *hopX1* ^C179A^ or an empty vector control on *Arabidopsis* Col-0 plants two days after spray inoculation with bacteria at 10^8^ cfu/ml^−1^ supplemented with 2 µM of COR or a mock solution. Error bars indicate standard error of the mean (SEM). Red asterisks indicate statistically significant values compared to *Pto* DC3000 *COR−* carrying an EV treated in each condition (mock or COR treated) (Student's *t* test, **p<0.01). The results are representative of three independent experiments. (**F**) HopX1 promotes bacterial growth on *Arabidopsis coi1* mutants. Growth of a COR-deficient *Pto* DC3000 strain expressing *hopX1*, *hopX1* ^C179A^, or an empty vector control on *Arabidopsis coi1-30* plants two days after spray inoculation as in (E) supplemented with 2 µM of COR or a mock solution. Error bars indicate SEM. Red asterisks indicate statistically significant differences compared to *Pto* DC3000 *COR-* carrying an EV treated in each condition (mock or COR treated) (Student's *t* test, **p<0.01). The results are representative of two independent experiments. (**G**) Infection of *Arabidopsis* plants with *Pto* DC3000 *COR−* carrying HopX1 triggers JAZ1ΔJas degradation. Immunoblots showing JAZ1ΔJas accumulation in transgenic Col-0 plants expressing a *JAZ1ΔJas-HA* transgene under the control of the *35S* promoter after mock treatment, or infection with *Pto* DC000, or *Pto* DC3000 COR− expressing *hopX1*, *hopX1* ^C179A^, or an empty vector. Non-transgenic Col-0 plants are included as a control. This experiment was repeated three times with similar results.

The results described above suggest that HopX1 could be responsible for, or at least participate in, the induction of JA-dependent responses by *Pta* 11528. To test if HopX1 is sufficient for this activation, we analyzed JA marker gene expression in stable transgenic *Arabidopsis* lines expressing *hopX1* or *hopX1* ^C179A^ genes from a *dexamethasone*-inducible promoter (*DEX*). These transgenic lines were constructed in *Arabidopsis* accession Aa–0, an ecotype that does not trigger cell death in response to certain *HopX1* alleles [38]. As JA markers, we chose genes induced early after JA treatment such as *JAZ5*, *JAZ10*, and *JAZ12*. As shown in Figures 4A and S10, induction of *hopX1* expression by DEX treatment for 36 hours strongly up-regulated all three *JAZ* marker genes in transgenic *hopX1 Arabidopsis*, whereas transcript levels remained low in transgenic plants expressing *hopX1* ^C179A^ or an EV control. Moreover, transgenic plants expressing *hopX1* developed chlorotic symptoms after DEX treatment, in contrast with *hopX1* ^C179A^ plants (Figure 4B and 4C). Development of chlorosis was correlated with losses in chlorophyll content (Figure S11), a hallmark response of the JA pathway [47]. Similarly, transient expression of *hopX1* by DEX treatment in *N. benthamiana* for 3 days, but not of *hopX1* ^C179A^ or an *EV* control, also induced strong chlorotic symptoms in the infiltrated area (Figure S12). Therefore, HopX1 triggers the activation of JA-dependent gene expression and JA-related phenotypes in a cysteine catalytic triad-dependent manner when ectopically expressed in *Arabidopsis*.

Crosstalk between JA and SA signaling pathways plays an important role in the regulation and fine-tuning of induced defenses against pathogens. The SA and JA/ET defense pathways generally antagonize each other, and induction of the JA pathway by *P. syringae* strains counteracts SA-dependent defenses [7]. Thus, we next determined whether activation of the JA-pathway by HopX1 could interfere with SA-dependent gene expression. We analyzed the expression of the SA marker gene *PR1* in *DEX*-inducible transgenic *Arabidopsis* Aa–0 plants expressing *hopX1 or hopX1* ^C179A^ pre-treated with 1 mM of SA or a mock solution for 24 hours. Treatment with SA strongly induced *PR1* expression in non-induced *hopX1* or *hopX1* ^C179A^ transgenic plants (Figure 4D). Strikingly, DEX induction of the *hopX1* effector gene for 24 hours strongly reduced subsequent SA induction of *PR-1* expression (Figure 4D). Pre-induction of the *hopX1* ^C179A^ gene interfered weakly with *PR-1* expression, but to a much lesser extent than wild-type HopX1. Overall, these results suggest that HopX1 activates the JA pathway to suppress SA-dependent defense responses to induce plant susceptibility.

On the basis of these results, we hypothesized that HopX1 could contribute to bacterial pathogenicity by mimicking COR-induced susceptibility. The jasmonate mimic COR is a bacterial toxin that contributes to bacterial invasion of the apoplast by *Pto* DC3000 [12]. Consistently, coronatine-deficient (*COR−*) *Pto* DC3000 mutants are less virulent on *Arabidopsis* plants when surface-inoculated [12]. To test if HopX1 contributes to bacterial pathogenicity during natural infections when delivered by the bacterial TTSS we compared bacterial replication of a *Pto* DC3000 *COR−* strain expressing *hopX1*_Pta11528_ or the *hopX1* ^C179A^ gene on *Arabidopsis* Col-0 plants infected by spray inoculation. This type of inoculation mimics natural infection conditions and is one of the most sensitive techniques to assess plant susceptibility to bacterial pathogens [48]. *Pto* DC3000 *COR−* growth was increased by about one log (cfu/cm^2^) when expressing *hopX1* compared to the same strain containing *hopX1* ^C179A^ or an empty vector construct (Figure 4E). Strikingly, differences in bacterial growth promoted by HopX1 were abolished when DC3000 *COR−* strains expressing *EV* or the *hopX1* or *hopX1* ^C179A^ genes were supplemented with 2 µM of COR (Figure 4E). This result supports the idea that HopX1 and COR act redundantly. Similar results were obtained when we compared bacterial replication on transgenic *Arabidopsis* Aa–0 plants expressing *hopX1* or *hopX1* ^C179A^ infected with *Pto* DC3000 or the isogenic *Pto* DC3000 *COR−* strain by spray inoculation (Figure S13). These results indicate that HopX1 can complement the deficiency in COR production of *Pto* DC3000 *COR−*, and supports a key role for the effector catalytic triad in the activation of the jasmonate pathway.

We further studied whether *Pto* DC3000 *COR−* growth promotion by HopX1 was COI1-dependent by performing similar experiments in *Arabidopsis* plants lacking the *COI1* receptor gene (*coi1-30*). As described previously, external application of COR did not restore *Pto* DC3000 *COR−* growth on *coi1-30* plants (Figure 4F) [10],[11]. In contrast, expression of *hopX1*, but not *hopX1* ^C179A^ or *EV*, enhanced the growth of *Pto* DC3000 *COR−* in *coi1-30* plants, similar to previous results in Col-0 plants (Figure 4F). Moreover, we examined whether JAZ proteins were degraded by HopX1 *in vivo* after bacterial infections on transgenic *Arabidopsis* Col-0 plants expressing the dominant negative J*AZ1ΔJas-HA* variant from the *35S* promoter. As described above, JAZ proteins lacking the C-terminal Jas domain do not interact with COI1 and cannot be ubiquitinated and degraded by the proteasome, thus promoting JA-insensitivity [17],[18],[21],[24],[46]. J*AZ1ΔJas-HA* levels were strongly reduced in total plant extracts 24 hours after infiltration of *Pto* DC3000 *COR−* bacteria expressing *hopX1*, whereas infiltration of buffer or *Pto* DC3000 *COR−* expressing either *hopX1* ^C179A^ or *EV* did not cause any significant changes (Figure 4G). Consistent with the fact that J*AZ1ΔJas-HA* is resistant to COI1-dependent degradation by bacterial COR, challenge with *Pto* DC3000 did not alter J*AZ1ΔJas-HA* levels (Figure 4G). Finally, we investigated whether *Pto* DC3000 *COR−* bacteria expressing *hopX1* could re-open plant stomata. To do this, we incubated *N. benthamiana* leaves with *Pto* DC3000 as a positive control or *Pto* DC3000 *COR−* bacteria expressing *hopX1*, *hopX1* ^C179A^, or an *EV* control. Incubation of *N. benthamiana* leaves with *Pto* DC3000 *COR−* expressing an *EV* for 5 hours induced stomatal closure whereas stomata of *N. benthamiana* leaves incubated with *Pto* DC3000 remained open similar to mock treated control leaves as it was described previously (Figure S14) [12]. Strikingly, only *Pto* DC3000 *COR−* bacteria expressing *hopX1*, but not *hopX1* ^C179A^, could re-open plant stomata of *N. benthamiana* leaves. This indicates that HopX1 from *Pta* 11528 plays a role in re-opening of stomata as COR does. These data are consistent with functional redundancy between HopX1 and the phytotoxin COR. Taken together, our data indicate that HopX1 acts inside the plant cell to promote activation of the JA pathway and induce susceptibility in *Arabidopsis*.

## Discussion

Levels of resistance in whole plants are influenced by systemic signals mediated, in many cases, by plant hormones. The importance of the role of hormones in biotic interactions is underlined by the increasing number of pathogenic microbes that are known to produce phytohormones or phytohormone mimics to perturb hormonal homeostasis and promote disease [6]. To date, production of cytokinins (CKs) [49], abscisic acid (ABA) [50], auxin [51], JA [52], and ET [53] has been reported in various bacterial or fungal species [6]. Remarkably, some pathogens produce hormone mimics. This is the case of some strains of *P. syringae* that produce COR, a mimic of the bioactive jasmonate JA-Ile, but synthesized via an unrelated biosynthetic pathway involving ligation of coronamic acid (cma) to the polyketide coronafacic acid (cfa) [2],[6]. Like JA-Ile, COR functions as an SA antagonist to promote virulence via suppression of host defenses. Notably, COR is produced by only a few *P. syringae* pathovars, whereas all *Pseudomonas* inject an array of effector proteins into the host cell that collectively promote disease by targeting and altering host cellular activities. The identification of their cellular targets is crucial to understand virulence. Recent data indicate that some effectors might impinge the JA pathway. For instance, Cui and colleagues showed that the effector AvrB indirectly perturbs JA signaling by interfering with the *Arabidopsis* mitogen-activated protein kinase MAP kinase 4 (MPK4) [35]. Moreover, several studies suggest that even in strains producing COR, effectors act synergistically with COR to induce the JA pathway [31]–[34]. Thus, it seems plausible that *Pseudomonas* manipulates the plant hormonal network through bacterial effectors to induce susceptibility. Here we have uncovered a novel molecular mechanism by which a bacterial strain (*Pta* 11528), which does not produce COR, activates the JA pathway to promote susceptibility through the degradation of JAZ repressors by the effector HopX1. In this work, we analyzed a *Pta* 11528 effector library containing ten identified effectors whereas the full *Pta* 11528 effector repertoire is predicted to secrete a suite of about 30 virulence effector proteins into the host cytoplasm [30]. Thus, it is plausible that additional effectors among the *Pta* 11528 effector repertoire not tested in this work may also destabilize JAZ proteins in a redundant manner with HopX1 to ensure activation of JA signaling and bacterial pathogenesis.

Expression of *hopX1*, but not *hopX1* ^C179A^, induced expression of early JA-responsive genes while reducing SA-mediated induction of the SA-marker gene *PR1*, a hallmark response of the JA pathway. These phenotypes are evidently associated with the virulence function of the effector, as the presence of HopX1 promotes *Pto* DC3000 growth both in stable transgenic *Arabidopsis* plants expressing the effector under the control of a inducible promoter, or when the effector is delivered naturally by the TTSS of the bacteria. These results highlight a novel bacterial strategy to subvert the antagonistic relationship between host SA and JA signaling pathways. JA-Ile activates plant responses by promoting physical interaction between the E3 ligase COI1 and JAZ repressors. This interaction leads to JAZ polyubiquitination (poly-Ub) and subsequent degradation by the 26S proteasome, releasing TFs from repression [16]–[18]. Remarkably, HopX1 compromises the accumulation of JAZ repressors in *NtCOI1*-silenced plants, and triggers the degradation of dominant-negative JAZ variants lacking the C-terminal Jas domain, which is required for the interaction with COI1 [17],[18],[26]. Therefore, HopX1-triggered degradation of JAZ proteins occurs in a COI1-independent manner, thus allowing activation of the pathway in the absence of the hormone. The ability of HopX1 to promote degradation of JAZ proteins and activate JA responses suggests that this is a strategy to promote disease. PD and co-immunoprecipitation experiments demonstrated that HopX1 interacts with JAZ proteins through its central ZIM domain, conserved in all 12 *Arabidopsis* JAZ repressors and their JAZΔJas variants. The interaction with the ZIM domain is consistent with HopX1-triggered degradation of all full-length JAZs and truncated derivatives tested in our assays, and indicates that the effect of HopX1 on JAZs is direct or at least through the same protein complex. JAZ proteins are translated in the cytoplasm and then localized mainly, but not exclusively, in the nucleus [17],[18],[54]. For example, JAZ1 can be detected in both cytosol and nucleus [55]. Moreover, Withers and colleagues recently reported a MYC2-dependent mechanism for nuclear import of cognate JAZs transcriptional repressors in *Arabidopsis* [54], which implied an equilibrium of JAZ proteins between the nucleus and the cytoplasm. Thus, it is conceivable that HopX1 could potentially degrade JAZ proteins both in the cytoplasm and nucleus.

Interaction and degradation of JAZs by HopX1 suggest that this effector has proteolytic activity. HopX1 family members are modular proteins that contain a putative cysteine-based catalytic triad and a novel conserved N-terminal domain [38]. The putative catalytic triad consists of cysteine (C), histidine (H), and aspartic acid (D) and is similar to that utilized by diverse enzyme families such as cysteine proteases and peptide N-glycanases (PNGases), which are all members of the transglutaminase (TGase) protein superfamily [38]. While previous reports failed to detect protease activity *in vitro* using general substrates, genetic approaches demonstrated the functional relevance of the conserved catalytic triad of HopX1 family members [38]. For example, mutation of this putative catalytic triad of various HopX family members abolished avirulence activity on *R2*-expressing bean cultivars and also prevented initiation of cell death in *Arabidopsis* following transient expression assays [38]. We found that HopX1 has protease activity on both general and specific substrates when the effector was directly immunopurified from plants and that this activity required Cys-179 within the conserved catalytic triad. Previously, Coaker and colleagues reported that the cysteine protease effector AvrRpt2 requires host general folding catalyst cyclophilins like *Arabidopsis* ROC1 to activate AvrRpt2 cysteine protease activity once the effector is translocated into the plant cell [56],[57]. Activated AvrRpt2 undergoes autoprocessing of its N-terminus to yield the mature protease that cleaves the host target RIN4 *in planta* [58]. Thus, it is possible that HopX1 also requires a host eukaryotic chaperone into the plant cell for the activation of the protease activity in a similar fashion as AvrRpt2. These results, together with the ability of HopX1 to interact with JAZs in PD and co-immunoprecipitation assays, suggest a model in which HopX1 acts on JAZs directly or indirectly through a third plant protein in the same complex. Noteworthy, HopX1^C179A^ shows markedly reduced affinity for JAZ proteins in PD and co-immunoprecipitation assays compared to wild-type HopX1 (Figures 3C, 3D, and S8). Thus, it is possible that the lack of activity of HopX1^C179A^ on JAZs could be due not exclusively to a missing enzymatic activity but also to a compromised ability to interact with its host targets, or a combination of both simultaneously. In this regard, we can also not completely exclude that HopX1 activity on JAZs could be due to an additional plant protease that co-purifies between the same protein complex. Despite previous reports showing that the cysteine protease effector AvrPphB cleaved host targets into smaller fragments [58],[59], we did not see smaller bands in our assays including free MBP. We speculate that this could be due to the small size of JAZ proteins (25–35 KDa). However, it is also possible that HopX1 degrades JAZ proteins at multiple sites and triggers target degradation in a similar fashion to the cysteine protease effector AvrRpt2 on its 30-kDa host substrate protein RIN4 [58],[59]. On the other hand, it is possible that the immunoprecipitation of HopX1-HA or C179A-HA from transgenic plants contains additional proteases that could affect MBP stability. This would explain why in Figure 2B MBP-JAZ5 protein purified from *E. coli* is more stable in the presence of general cocktail protease inhibitors. Despite this, unspecific degradation would occur in all samples to the same extent and therefore, the effect we observed in MBP-JAZ5 in the presence of HopX1 is likely to be specific. Moreover, we found that substitution of the conserved Cys-179 amino acid of HopX1 to alanine also compromised HopX1-mediated JAZ5 degradation *in vivo*. Similarly, activation of JA-dependent gene expression and JA-related phenotypes occurred in a Cys-179 dependent manner when expressed ectopically in *Arabidopsis*. Furthermore, in contrast to wild-type HopX1, HopX1^C179A^ delivered by the TTSS was unable promote growth of *Pto* DC3000 *COR−* bacteria. Overall, these data suggest that HopX1 family members are proteolytic enzymes of the cysteine protease family, and that this activity is required for function.

Several JAZ pre-mRNAs are subject to alternative splicing, which, at least for JAZ10, results in truncated JAZΔJas proteins that are resistant to proteasomal degradation because they are unable to interact with COI1, thereby causing dominant JA-insensitive phenotypes [26],[45],[60]. The alternative splicing of *JAZ* genes provides a feedback mechanism to efficiently repress the JA signal output and reduce the fitness costs associated with over-stimulation of the signaling pathway [45],[60]. Degradation of JAZΔJas variants by HopX1 suggests that this effector should promote a sustained activation of the JA pathway, favoring susceptibility. In agreement with this hypothesis, *Pto* DC3000 COR− expressing *hopX1* promoted bacterial replication in *Arabidopsis* plants (Figure 4E and 4F). Moreover, *Pto* DC3000 COR−, which does not produce COR, grew better in the presence of the effector (DEX in Figure S13) than the wild-type strain producing COR in the absence of the effector (mock in Figure S13). Therefore, our results suggest that evolution has shaped this effector–host interaction to maximize the activation of the JA response pathway through direct targeting of most forms of JAZs. Besides the 12 JAZ repressors, six additional *Arabidopsis* proteins contain a ZIM domain. Collectively, these 18 proteins are known as the TIFY proteins [23],[61]. It remains to be determined whether HopX1 targets any of these ZIM domain containing proteins, and if they also play a role in plant defense against bacteria.

Notably, HopX homologs are found in diverse phytopathogenic bacteria including *Pto* DC3000. BLAST analysis revealed a near identical match of HopX1*_Pta_*_ 11528_ with HopX1 from *P. syringae* pv. *phaseolicola* bacterial strains such as race 4 (*Pph* race 4) (99% identity) but significantly less homology with the respective *Pto* DC3000 homolog (72% identity) (Figure S4A). Several lines of evidence suggest that the *Pto* DC3000 and *Pta* 11528 alleles of HopX1 play different roles in the plant cell. Firstly, HopX1*_Pto_*_ DC3000_ triggers cell death in several *Arabidopsis* ecotypes including Col-0 and Ws-0 whereas HopX1*_Pph_*_ race 4_ (which is almost identical to the *Pta* 11528 allele) does not [38]. Secondly, HopX1*_Pto_*_ DC3000_ did not compromise the accumulation of JAZ5 when co-expressed transiently in *N. benthamiana* (Figure S15). Consistently, transgenic *Arabidopsis* Col-0 plants expressing the dominant negative J*AZ1ΔJas* variant infected with *Pto* DC3000 did not alter J*AZ1ΔJas* levels (Figure 4G). This indicates that these plants are resistant to both HopX1*_Pto_*_ DC3000_ and COI1-dependent degradation by bacterial COR. The ability of HopX1*_Pta_*_ 11528_ to trigger JAZ degradation and activation of JA responses may reflect specialized functions among the broad divergence across the HopX family members. It is tempting to speculate that redundancy between COR and HopX1 has allowed strains that produce the phytotoxin to evolve HopX1 for other functions and in doing so, lose its JA-inducing activity. Overall, HopX1 exemplifies a bacterial strategy for pathogenicity in which the JA pathway is targeted through direct degradation of JAZ repressors to promote pathogenesis.

Finally, emerging data suggest that bacterial pathogens have evolved effectors to manipulate important plant hormones that regulate defense response such as SA and JA [6]. For, example, the *P. syringae* effector HopI1 directly targets Hsp70 in choloroplasts to suppress SA accumulation [62] whereas the effector AvrB promotes JA signaling likely through an indirect mechanism via the activation of MPK4 [35]. Very recently, the *P. syringae* effector HopZ1a was reported to interact and acetylate several JAZ proteins through a putative acetyltransferase activity [55]. HopZ1a-mediated acetylation induces JAZ1 degradation through an undefined mechanism that is dependent on COI1. This leads to activation of JA-dependent gene expression and plant susceptibility [55]. It is possible that post-translational modifications of JAZ1 induces or represses 26S proteasome degradation triggered by COI1. In this study, we found that *hopX1* encodes a cysteine protease, activity that is required for degradation of JAZs by HopX1. HopX1 associates with JAZ proteins through its central ZIM domain and degradation occurs in a proteasome- and COI1-independent manner, highlighting the different strategies used by bacterial effectors to target similar host components. Importantly, ectopic expression of HopX1 in *Arabidopsis* induces the expression of JA-dependent genes and represses SA-induced markers, whereas delivery of HopX1 by the natural TTSS of *Pseudomonas* partially re-opens stomata during the infection process and promotes susceptibility to a similar extent as the addition of COR. These results highlight a novel molecular mechanism by which bacterial effectors directly manipulates core regulators of hormone signaling to facilitate infection. Indeed, oomycete pathogens were also found to produce effectors that interact with JAZ3 [36]. The recent findings highlight the JA receptor complex as a major common and critical hub of suppression for diverse pathogens during the arms race between plants and pathogens.

## Materials and Methods

### Plant Materials and Growth Conditions

*Arabidopsis* Aa–0 plants were transformed as described previously [63] using the binary vector pTA7002 [64] containing the *hopX1* or the *hopX1* ^C179A^ gene fused to a C-terminal HA under the control of a *dexamethasome* (*DEX*)-inducible promoter. Homozygous single-insertion transformants were selected in the T3 generation and HopX1 or HopX1^C179A^ accumulation was confirmed by Western blots after induction with 30 µM DEX with 0.01% Silwet L-77. To generate transgenic plants expressing JAZ1ΔJas-HA in Col-0 background, JAZ1ΔJas was amplified with Expand High Fidelity polymerase (Roche) using Gateway-compatible primers (*AtJAZ1 ΔJas*, 5′-ggggacaagtttgtacaaaaaagcaggcttcATGTCGAGTTCTATGGAATGTTCTG-3′ and 5′-ggggaccactttgtacaagaaagctgggtcCTTGGCTAAGCTATTAGCGGT-3′). PCR products were cloned into pDONR207 with a Gateway BP II kit (Invitrogen) and sequence verified. This plasmid, a Gateway LR II kit (Invitrogen), and the pGWB14 [65] destination vector was used to generate *35S:JAZ1ΔJas-HA*. These constructs were transferred to *Agrobacterium tumefaciens* strain C58C1 by freeze thawing and then transformed in Col-0 plants by floral dipping method [66]. Hygromycin-resistant plants were selected and their T2 progenies propagated for subsequent analysis. The knockout line *coi1-30* [67] and stable *N. tabacum* lines silenced for an EV (line VC) or the *NtCOI1* gene (line18) [42] were previously described.

*N. benthamiana* and *N. tabacum* plants were grown in controlled environment chambers at an average temperature of 24°C (range 18°C–26°C), with 45%–65% relative humidity under long day conditions (16 h light). In contrast, *A. thaliana* plants were grown in controlled environment chambers at an average temperature of 22°C (range 16°C–24°C), with 45%–65% relative humidity under short day conditions (10 h light). *N. benthamiana* and *N. tabacum* plants were grown for three to four weeks prior to *A. tumefaciens*-mediated transformation. *Arabidopsis* plants of four to six weeks were usually analyzed in bacterial growth assays.

### Bacterial Strains

The *E. coli* strain DH5α was used for cloning, and small- and large-scale plasmid isolation. *A. tumefaciens* C58C1 was used for transient expression in *N. benthamiana. Pseudomonas* strains used in this study were *Pseudomonas syringae* pv. *tomato* (*Pto*) DC3000, *Pseudomonas syringae* pv. *tabaci* (*Pta*) 11528, a non-pathogenic TTSS defective *Pta* 11528 *hrpV−* bacteria unable to secrete effector proteins into the plant cell [37] and the coronatine-deficient *Pto* DC3000 strain (*Pto* DC3000 *COR−*) which is a *Pto* DC3000 AK87 mutant that carries mutations in cmaA (coronamic acid A) and cfa6 (coronafacic acid 6) [11]. HopX1 or HopX1^C179A^ were cloned into pCPP5040, a derivative of the broad-host-range vector pML123 [68], which expresses insert genes from the *nptII* promoter, and generates protein products for expression in *Pseudomonas* species [41]. This vector was a kind gift from Emilia Lopez Solanilla and Pablo Rodriguez Palenzuela. These plasmids were introduced into *Pto* DC3000 *COR−* by triparental mating in which pRK600 was used as a helper plasmid.

### Stomatal Aperture Measurements

Leaf discs from 4- to 5-week-old *N. benthamiana* young leaves were exposed to white light for 1 hour while submerged in a solution containing 50 mM KCl, 10 µm CaCl_2_, and 10 mm MES-KOH, (pH = 6.1) to induce stomatal aperture. Subsequently, leaf discs were immersed in buffer or bacterial suspension at 5×10^8^ cfu/ml (optical density at 600 nm [OD_600_] = 1) in 50 mM KCl, 10 µm CaCl_2_, and 10 mm MES-KOH (pH = 6.1). The samples were incubated under the same conditions for 5 hours. Abaxial leaf surfaces were observed with a microscope (Leica DMR), and stomatal aperture was measured using ImageJ software.

### Statistical Methods

Statistical significance based on *t* test analysis was developed by GraphPad Prism program. Seven independent samples were used to analyze the significance of bacterial growth results.

Seventeen independent stomata were used to analyze stomatal aperture in each condition.

### Transient Expression Assays and Inhibitory Assays

Growth and transient expression conditions were as described previously [69] using the *A. tumefaciens* strain C58C1. For transient gene expression, *A. tumefaciens* was syringe infiltrated in *N. benthamiana* or *N. tabacum* leaves at OD_600_ = 0.4, whereas for transient gene co-expression assays both constructs were infiltrated at OD_600_ = 0.3. Samples were collected after two days. The genes expressed in this paper were *35S:hopX1-HA, DEX:hopX1-HA*, *DEX:hopX1* ^C179A^-*HA*, *35S:GFP, 35S:GFP-hopX1*, *35S:GFP-hopX1* ^C179A^, *35S:JAZ-HA* (*Arabidopsis* cDNA coding sequences cloned in constructs for all 12 JAZs), *35S:COI1-GFP, 35S:MYC2-HA, 35S:JAZ1ΔJas-HA, 35S:JAZ2ΔJas-HA*, and *35S:JAZ7ΔJas-HA*. We also used the *Pto* DC3000 effector genes *35S:hopX1_Pto_*_ DC3000_-*HA*, *35S:HopC1_Pto_*_ DC3000_-*HA*, *35S:HopAD1_Pto_*_ DC3000_-*HA*, and *35S:HopN1_Pto_*_ DC3000_-*HA*. For inhibitory assays *in planta*, MG132 (Sigma) inhibitor was co-infiltrated with *A. tumefaciens* at a concentration of 100 µM.

### Protein Extraction and Immunoblotting

Total proteins were extracted from leaf tissue by homogenization in extraction buffer (100 mM Tris-HCl [pH 7.5], 150 mM NaCl, 5 mM EDTA, 5% glycerol, 10 mM DTT, 2% PVPP, 1 mM PMSF, protease inhibitors cocktail [Roche], and 0.5% Triton X-100). In all experiments protein samples were equilibrated to equivalent concentrations of total proteins. Extracted proteins were fractioned by 8%–10% SDS-PAGE, transferred onto HybondTM-P membranes (Amersham) and incubated with anti-HA-horseradish peroxidase (Roche) or anti-GFP-horseradish peroxidase antibody (Milteny Biotec). Immunodetection was performed with ECL chemiluminiscence reagent (GE Healthcare) or Supersignal West Femto (Thermo Scientific).

### Pull-Down Assays

MBP-JAZ fusion proteins were generated as previously described [21]. Ten-day-old *Arabidopsis* wild-type Col-0 seedlings and lines expressing *DEX:hopX1-HA* or *DEX:hopX1* ^C179A^-*HA* were induced with 30 µM DEX plus 0.01% Silwet L-77 or a mock solution for 24 hours. Seedlings were ground in liquid nitrogen and homogenized in extraction buffer containing 50 mM Tris-HCl, (pH 7.4), 150 mM NaCl, 10% glycerol, 0.2% NP-40, 1 mM DTT, 1 mM phenylmethylsulphonyl fluoride, 5 mM MgC_l2_, 50 mM MG132 (Sigma-Aldrich), and complete protease inhibitor (Roche). After centrifugation (16,000 *g* at 4°C), the supernatant was collected. For in vivo PD experiments, 6 µg of resin-bound MBP fusion protein was added to 250 µg of pre-equilibrated total protein extract and incubated for 1 h at 4°C with rotation. After washing, samples were denaturalized, loaded on 8% SDS-PAGE gels, transferred to nitrocellulose membranes, and incubated with anti-HA-horseradish peroxidase (Roche). A 5 µl aliquot of MBP-fused protein of each sample was run into SDS-PAGE gels and stained with Coomassie brilliant blue to confirm equal protein loading.

### Protease Activity Assays

Immunoprecipitation of HopX1-HA and HopX1^C179A^-HA from stable transgenic *Arabidopsis* plants using the anti-HA affinity matrix (Roche) was performed according to the manufacturer's instructions with some modifications (Anti-HA Affinity Matrix, catalogue number 11 815 016 001, Roche). Briefly, transgenic *Arabidopsis* seedlings expressing *hopX1-HA* and *hopX1* ^C179A^-HA or wild-type Col-0 used as a negative control were induced by spraying with 30 µM DEX plus 0.01% Silwet L-77 for 24 hours. The material was ground in liquid nitrogen and homogenized in extraction buffer containing 100 mM Tris-HCl (pH 7.5), 150 mM NaCl, 5 mM EDTA, 5% glycerol, 10 mM DTT, 2% PVPP, 1 mM PMSF, protease inhibitors cocktail (Roche), and 0.5% Triton X-100. The samples were centrifuged twice at 16,000 *g* at 4°C. The supernatant was incubated for 5 hours (4°C, with rotation) with the anti-HA affinity matrix (Roche), and then washed once with 1 ml of extraction buffer and three times with 1 ml of cool TBS (50 mM Tris-HCl [pH 7.5], 150 mM NaCl, [pH = 7.4]) to remove all protease inhibitors from the anti-HA affinity matrix. Elution of HA proteins from the anti-HA affinity matrix was performed by incubating with 1 mg/ml of HA peptide (Roche) in TBS buffer for 30 minutes at 37°C with strong shaking. Supernatant was recovered by centrifugation and transferred to a fresh tube for protease activity assays. Immunoprecipitated HopX1-HA and HopX1^C179A^-HA was pre-equilibrated for similar amounts of protein and maintained at 4°C until used.

In vitro Protease Fluorescent Detection kit to determine protease activity was performed according to the manufacturer's instructions (PF0100-1KT, Sigma). Briefly, 10 µl of immunoprecipitated HopX1-HA or HopX1^C179A^-HA (or MBP-HopX1 for Figure S4B) was incubated with 20 µl of Incubation buffer and 20 µl of fluorescein isothiocyanate (FITC)-casein substrate overnight in the dark at 37°C with moderate shaking. The reaction was stopped by treatment with 150 µl of 10% trichloroacetic acid (TCA) for 30 min at 37°C in the dark. Samples were then centrifuged, and supernatant was recorded for fluorescence intensity with excitation at 485 nm and monitored for the emission wavelength of 535 nm (485/535 nm).

For *in vitro* experiments regarding protease activity of HopX1 on JAZs, we incubated 20 µl of immunoprecipitated HopX1-HA or HopX1^C179A^-HA with 5 µl of recombinant JAZ5 fused to MBP expressed and purified from *E. coli* cells in a total volume of 50 µl TBS (with or without general protease inhibitors, Roche). The reaction was incubated overnight at 37°C with moderate shaking. To stop the reactions all samples were denaturalized, and then loaded on 10% SDS-PAGE gel, transferred to a nitrocellulose membrane, and incubated with anti-MBP (AbCAM). Immunodetection was performed with ECL chemiluminiscence reagent (GE Healthcare)

### Confocal Microscopy

HopX1 was placed into pENTR/D-TOPO (Invitrogen) using the 5′-ggggacaagtttgtacaaaaaagcaggcttcATGAGAATTCACAGTGCTGGTCA-3′ and 5′-ggggaccactttgtacaagaaagctgggtcTTATCTTCGTGGAGGCATGCCTTTAGACG-3′ primers, and then recombined into the gateway binary vector pGWB6 to create constructs that express N-terminal GFP fusion proteins. This was then transiently expressed in *N. benthamiana* leaves by *Agrobacterium*-mediated transformation. Localization of GFP fusion was visualized with sequential laser scanning confocal microscopy, using a Leica Confocal SP5 with sequential imaging at 488 nm excitation and 505–525 nm emission (green/GFP) and 633 nm excitation and 660 nm emission (red/chlorophyll).

### Plant Cell Fractionation

Plant cell fractionation was performed by using the CelLytic PN Isolation/Extraction kit for plant leaves (CELLYTPN1-1KT, Sigma) according to the manufacturer's instructions.

### Measure of Chlorophyll Content

The chlorophyll content of leaves was measured by acetone extraction according to Arnon [70]. Briefly, 100–150 mg tissue per sample was extracted for two hours in 400 µl of acetone 80% (v/v) at 4°C in the dark with constant shacking. The homogenate was centrifuged at 3,000 rpm for 2 minutes. The supernatant was saved (V_1_) and 0.2 ml of the supernatants were diluted in a known volume of acetone 80% until the absorbance of the extract at 663 nm and 645 nm was read between 0.2–0.8 (V_2_) using a SpectraMax Absorbance Microplate Reader. The concentration of chlorophyll a (C_a_), b (C_b_), and total chlorophyll (C_t_) was calculated using Arnon's equations: C_a_ = 12.7 * A_663_−2.63 * A_645_, C_b_ = 22.9 * A_645_−4.68 * A_663_, and C_t_ = C_a_+C_b_ * [V1 * (V2+0.2)/(0.2 * P]. Ca, Cb are expressed in µg * ml^−1^, P in grams of fresh leave tissue, V in ml, and C_t_ in µg * ml^−1^ of fresh weight.

### Quantitative RT-PCR

Quantitative RT-PCR for JA-dependent gene expression experiments were performed with RNA extracted from 10-day-old seedlings grown on liquid MS media that were treated with 5 µM DEX for 36 h or a mock solution. For quantitative RT-PCR analysis of *PR1* expression in stable transgenic *Arabidopsis* lines expressing the *hopX1* or *hopX1* ^C179A^ genes, 10-day-old seedlings grown on liquid MS media were DEX-induced for 24 hours and then treated with 1 mM SA or a mock solution for additional 24 hours. For each experiment, three biological replicates, consisting of tissue pooled from 15 to 20 plants were taken. For semi-quantitative RT-PCR of AtJAZ5 expression levels when co-expressed with HopX1 in *N. benthamiana*, discs from six independent leaves were collected and frozen in liquid nitrogen two days after infiltration. For semi-quantitative RT-PCR analysis of stable *NtCOI1*-silenced *N. tabacum* plants, discs from six independent leaves of four weeks old plants were collected and frozen in liquid.

RNA extraction and cleanup was done using Trizol reagent (Invitrogen) followed by RNeasy mini kit (Qiagen) and DNase digestion to remove genomic DNA contamination. cDNA was synthesized from 0.5 to 1 µg of total RNA with the high-capacity cDNA reverse transcription kit (Applied Biosystems). Two microliters from one-tenth diluted cDNA was used to amplify selected genes. For quantitative PCR analysis, Power SYBR Green was used for gene amplification (Applied Biosystems). Quantitative PCR was performed in 96-well optical plates in a 7300 Real Time PCR system (Applied Biosystems). Data analysis shown was done using three technical replicates from one biological sample; similar results were obtained with two additional independent biological replicates.

Primers for genes used here are as follows: *AtJAZ10*, 5′-GAGAAGCGCAAGGAGAGATTAG-3′ and 5′-CTTAGTAGGTAACGTAATCTCC-3′; *AtJAZ5*, 5′-AAAGATGTTGCTGACCTCAGTG -3′ and 5′-CCCTCCGAAGAATATGGTCA-3′; *AtJAZ*12, 5′-CATCTAATGTGGCATCACCAG-3′ and 5′-TGCCTCCTTGCAATAGGTAGA-3′; *AtPR1*, 5′-AAGTCAGTGAGACTCGCATGTGC-3′ and 5′-GGCTTCTCGTTCACATAATTCCC-3′; *AtACTIN8*, 5′-CCAGTGGTCGTACAACCGGTA -3′ and 5′- TAGTTCTTTTCGATGGAGGAGCTG-3′; *HopX1*, 5′-TAGCAAGCTTCGCTTACG-3′ and 5′-GTTTCACGCGTAACCTTG-3′; *NtCOI1*, 5′-GAAGATCTTGAATTGATGGC-3′ and 5′-CCCAGAAGCATCCATCTCAC-3′; and *NtαTubulin*, 5′-AGTTGGAGGAGGTGATGATG-3′ and 5′-TATGTGGGTCGCTCAATGTC-3′.

### Bacterial Growth Curves

Transgenic *Arabidopsis* plants expressing *hopX1* and *hopX1* ^C179A^ were induced by spraying with 30 µM DEX plus 0.01% Silwet L-77 or a mock solution for 24 hours, and then infected with selected bacteria. All bacterial growth assays in *Arabidopsis* were performed by spray inoculation as described [48]. Briefly, overnight bacterial cultures were pelleted and resuspended in sterile 10 mM MgCl_2_. Plants were sprayed with a bacterial suspension containing 10^8^ (cfu)/ml bacteria (OD_600_ = 0.2) with 0.04% Silwet L-77. Leaf discs were harvested after two days and ground in 10 mM MgCl_2_. Serial dilutions of leaf extracts were plated on LB agar with appropriate antibiotics. Each data point represents the average of seven replicates, each containing two leaf discs from different plants. Error bars indicate standard errors of the mean (SEM). These experiments were repeated at least three times with similar results, and representative results are shown.

Immunoblots showing JAZ1ΔJas degradation in *Pto* DC3000 COR− expressing *hopX1* infected Col-0 *Arabidopsis* plants were performed after syringae infiltration of the indicated bacterial suspension at 10^8^ (cfu)/ml (OD_600_ = 0.2) into the leaves. Samples were collected after 24 hours for protein analysis.

## Supporting Information

Figure S1 **Live** ***Pta*** **11528 bacteria manipulate stomatal aperture in a TTSS-dependent manner.** Stomatal aperture in wild-type *N. benthamiana* leaves measured after 5 hours of incubation with mock or bacterial strains *Pto* DC3000, *Pta* 11528, or *Pta* 11528 *hrpV−*. Error bars indicate SEM (*n* = 17). Asterisks indicate significant differences compared with mock-treated samples at **p<0.01. The results are representative of three independent experiments.
(TIF)

Figure S2 **HopX1 compromises the accumulation of JAZ5.** Immunoblots showing JAZ5-HA accumulation in the presence of HopX1-HA when co-expressed transiently in *N. benthamiana* for two days. Proteins were detected with anti-HA antisera. CBB, Coomassie brilliant blue staining. The results are representative of three independent experiments.
(TIF)

Figure S3 **Phylogenetic tree of the** ***Arabidopsis*** **JAZ proteins.** Phenogram representation of the neighbor-joining phylogenetic tree of the 12 full-length JAZ proteins. The sequence alignment was generated using DiAlign (Genomatix) and the tree was created by Phylodendron (University of Indiana). Branch lengths are proportional to the estimated evolutionary distance. Bootstrap values are included. JAZ proteins can be tentatively grouped into four clades.
(TIF)

Figure S4 **HopX family members contain a consensus cysteine-based catalytic triad and a conserved N-terminal domain.** (A) HopX family members contain a consensus cysteine-based catalytic triad and a conserved N-terminal domain. Conservation of the putative catalytic residues (red dots) and the N-terminal domain (red line) between *P. syringae* HopX1 alleles from *Pta* 11528, *Pto* DC3000, and *Pph* race 4. Sequences were downloaded from the National Center for Biotechnology Information and were aligned using DiAlign (Genomatix). (B) MBP-HopX1 has no protease activity *in vitro* on the general substrate casein. Protease activity of recombinant MBP-HopX1 or MBP-HopX1^C179A^ incubated with fluorescein isothiocyanate (FITC)-labeled casein at 37°C overnight with moderate shaking. Trypsin was used as a positive control. Fluorescence units record the fluorescence intensity with excitation at 485 nm and monitor the emission wavelength of 535 nm (485/535 nm). An immunoblot showing MBP-HopX1 and MBP-HopX1^C179A^-HA effector inputs is also shown. This experiment was repeated three times with similar results. (C) Degradation of JAZ1, JAZ2, JAZ9 and JAZ10 by HopX1 requires the cysteine-based catalytic triad of a putative protease *in vivo*. The immunoblots show JAZ1-HA, JAZ2-HA, JAZ9-HA, JAZ10-HA and MYC2-HA accumulation in the presence of GFP-HopX1 or GFP-HopX1^C179A^ when co-expressed transiently in *N. benthamiana*. This experiment was repeated twice with similar results.
(TIF)

Figure S5 **The cysteine proteases HopC1 and HopN1, or the unrelated effector HopAD1-HA, do not compromise JAZ accumulation.** (A) Immunoblots showing JAZ5-HA accumulation in the presence of HopX1-HA, the cysteine protease HopC1-HA or an empty vector control when co-expressed transiently in *N. benthamiana* for 2 days. Proteins were detected with anti-HA antisera. CBB, Coomassie brilliant blue staining. This experiment was repeated twice with similar results. (B) Immunoblots showing JAZ5-HA accumulation in the presence of HopX1-HA, the cysteine protease HopN1-HA, the unrelated effector HopAD1-HA, or an empty vector control when co-expressed transiently in *N. benthamiana* for 2 days. Proteins were detected with anti-HA antisera. CBB is shown. This experiment was repeated three times with similar results.
(TIF)

Figure S6 **JAZ5 degradation by HopX1 is independent of the 26S proteasome.** Immunoblots showing JAZ5-HA accumulation in the presence of GFP-HopX1 or an empty vector control when co-expressed transiently in *N. benthamiana* for 2 days after treatment with 100 µM of MG132. This experiment was repeated twice with similar results.
(TIF)

Figure S7 **Molecular and phenotypic analysis of** ***NtCOI1*** **-silenced** ***N. tabacum*** **plants.** (A) RT-PCR of *NtCOI1* expression in leaves of *N. tabacum*-silenced EV plants (Line VC) or *N. tabacum* plants silenced for the *NtCOI*1 gene (Line L18). *N. tabacum α-Tubulin* (*NtTUB*) was used as an amplification control. This supporting figure is related to Figure 3A. (B) Capsule and seeds production obtained from transgenic *N. tabacum* plants silenced with an EV construct (Line VC) or *NtCOI1* (Line 18).
(TIF)

Figure S8 **MBP-HopX1 co-immunoprecipitates with JAZ5-GFP from** ***N. benthamiana*** **plant extracts.** MBP-HopX1 and MBP- HopX1^C179A^ proteins purified from *E. coli* cells were incubated 2 hours with *N. benthamiana* plants extracts transiently expressing the JAZ5-GFP transgene and then subjected to co-immunoprecipitation analysis using GFP agarose beads. CBB, Coomassie brilliant blue staining. This experiment was repeated twice with similar results.
(TIF)

Figure S9 **HopX1 subcellular localization in plant cells.** (A) Confocal microscopy localization of GFP- HopX1 or GFP alone in transiently transformed *N. benthamiana* leaves 48 hours post-infiltration (green). The nuclear GFP signal of a middle cross section is shown. This supporting figure is related to Figure 3F. Note that the pictures show confocal sections of the nuclei and, therefore, the signal is nuclear and not derived from GFP protein surrounding the nuclear membrane. (B) Crude subcellular fractionation of GFP-HopX1 in *N. benthamiana*. Immunoblots showing GFP-HopX1 localization after subcellular fractionation when GFP-HopX1 or GFP alone was expressed transiently in *N. benthamiana* leaves for 48 hours. Total, cytoplasmic and nuclear fractions are shown. Actin, cytoplasmic intrinsic protein for control of the purity of the nuclear fraction. RNA polymerase II, nuclear intrinsic protein for control of enrichment of the nuclear fraction. Equivalent fraction volumes were loaded. This experiment was repeated twice with similar results.
(TIF)

Figure S10 **HopX1 triggers the activation of JA-dependent gene expression in** ***Arabidopsis*** **.** Quantitative RT-PCR analysis of *JAZ5* and *JAZ12* expression on Col-0 (EV) and stable transgenic *Arabidopsis* Aa–0 lines expressing the *hopX1* or *hopX1* ^C179A^ genes 36 hours after treatment with DEX or a mock solution. The measurements (three technical replicates) represent the relative expression levels between mock (control) and DEX-treated plants in each *Arabidopsis* background. All samples were normalized against the housekeeping gene *AtACT8*. Error bars represent standard deviation (SD). The results are representative of three independent experiments.
(TIF)

Figure S11 **Ectopic expression of** ***hopX1*** **but not** ***hopX1*** **^C179A^** **in stable transgenic** ***Arabidopsis*** **Aa–0 correlates with loss in chlorophyll content.** (A) Contents of leaf chlorophyll in stable transgenic *Arabidopsis* lines expressing the *hopX1* or *hopX1* ^C179A^ genes two, five, and nine days after DEX treatment. Error bars represent standard deviation (SD). L12 and L13 are two independent stable transgenic *Arabidopsis* lines expressing the *hopX1* gene. L15 and L16 are two independent stable transgenic *Arabidopsis* lines expressing the *hopX1* ^C179A^ gene. The results are representative of three independent experiments. (B) Immunoblots showing HopX1-HA or HopX^1C179A^-HA accumulation in stable *Arabidopsis* lines expressing the *hopX1* or *hopX1* ^C179A^ genes. Plants were induced with DEX or a mock solution for five hours. Line designations are as for Figure S11A.
(TIF)

Figure S12 **HopX1 induces chlorosis in** ***N. benthamiana*** **leaves when transiently expressed.** Chlorotic symptoms in transiently expressed *EV*, *hopX1*, or *hopX1* ^C179A^ *N. benthamiana* leaf tissue after DEX treatment for 48 hours.
(TIF)

Figure S13 **HopX1 complements the growth of a COR-deficient** ***Pto*** **DC3000 strain when expressed ectopically in** ***Arabidopsis*** **Aa–0 plants.** Growth of *Pto* DC3000 and the isogenic *Pto* DC3000 *COR−* strain on stable transgenic *Arabidopsis* lines expressing the *hopX1* or *hopX1* ^C179A^ genes two days after spray inoculation with bacteria at 10^8^ cfu/ml^−1^. Plants were pre-treated with DEX or a mock solution 24 hours prior to bacterial infection. Pto DC3000 growth increased by about one log (cfu/cm^2^) in transgenic HopX1 *Arabidopsis* pre-treated with DEX compared to mock treated control plants whereas ectopic expression of *hopX1* ^C179A^ could only promote Pto DC3000 growth to about half a log (cfu/cm^2^). The data indicate that HopX1 contributes to pathogenicity over 2-fold compared to HopX1C179A when effectors are ectopically overexpressed in *Arabidopsis* plants. Furthermore, in mock treated transgenic HopX1 *Arabidopsis* plants, Pto DC3000 COR− growth was restricted by one log (cfu/cm^2^) compared to Pto DC3000. However, both strains grew to similar levels when expressing *hopX1* in response to DEX treatment. In contrast, Pto DC3000 COR− growth returned to wild-type levels when HopX1 carried the C179A mutation. Error bars indicate standard error of the mean (SEM). Red asterisks indicate statistically significant differences between DEX and mock-treated plants in each *Arabidopsis* line/bacterial strain (Student's *t* test, *p<0.05 and **p<0.01). Black asterisks indicate statistically significant differences between *Pto* DC3000 and *Pto* DC3000 *COR−* in each condition (Student's *t* test, *p<0.01). The results are representative of three independent experiments.
(TIF)

Figure S14 ***Pto*** **DC3000** ***COR−*** **bacteria expressing** ***hopX1*** **manipulate stomatal aperture.** Stomatal aperture in wild-type *N. benthamiana* leaves measured after 5 hours of incubation with mock, *Pto* DC3000, or *Pto* DC3000 *COR−* bacteria expressing *hopX1*, *hopX1* ^C179A^, or an *EV* control. Error bars indicate SEM (*n* = 20). Asterisks indicate significant differences compared with *Pto* DC3000 *COR−* bacteria expressing an *EV* control at **p<0.01. The results are representative of two independent experiments.
(TIF)

Figure S15 **HopX1** ***_Pto_***_** DC3000**_ **does not compromise the accumulation of JAZs when transiently co-expressed in** ***N. benthamiana*** **.** Immunoblots showing JAZ5-HA and JAZ10-HA accumulation in the presence of HopX1*_Pta_*_ 11528_, HopX1*_Pto_*_ DC3000_, or an empty vector control when co-expressed transiently in *N. benthamiana*. Proteins were detected with anti-HA. CBB, Coomassie brilliant blue staining. A non-specific band is shown as an additional loading control in the blot of JAZ10. This experiment was repeated twice with similar results.
(TIF)

Table S1 **List of** ***Pta*** **11528 secreted effector proteins included in this study.** Putative activities of type III effectors are shown. The *Pta* 11528 effector identity was determined by BLAST against all known effector proteins.
(TIF)

## Abbreviations

COR: coronatine
cfu: colony forming units
DEX: dexamethasome
ET: ethylene
EV: empty vector
GFP: green fluorescent protein
HA: hemagglutinin
JA: jasmonic acid
JA-Ile: jasmonate-Isoleucine
MBP: maltose binding protein
PD: pull down
*Pta*: *Pseudomonas syringae* pv. *tabaci*
*Pto* DC3000: *P. syringae* pv. tomato stain DC3000
SA: salicylic acid
TF: transcription factor
TTSS: type III secretion system
