# Supplementary figures and images for: The Bacterial Effector HopX1 Targets JAZ Transcriptional Repressors to Activate Jasmonate Signaling and Promote Infection in Arabidopsis
Source: PLoS Biol. 2014 Feb 18;12(2):e1001792. doi: 10.1371/journal.pbio.1001792 (PMC3928049; doi:10.1371/journal.pbio.1001792)

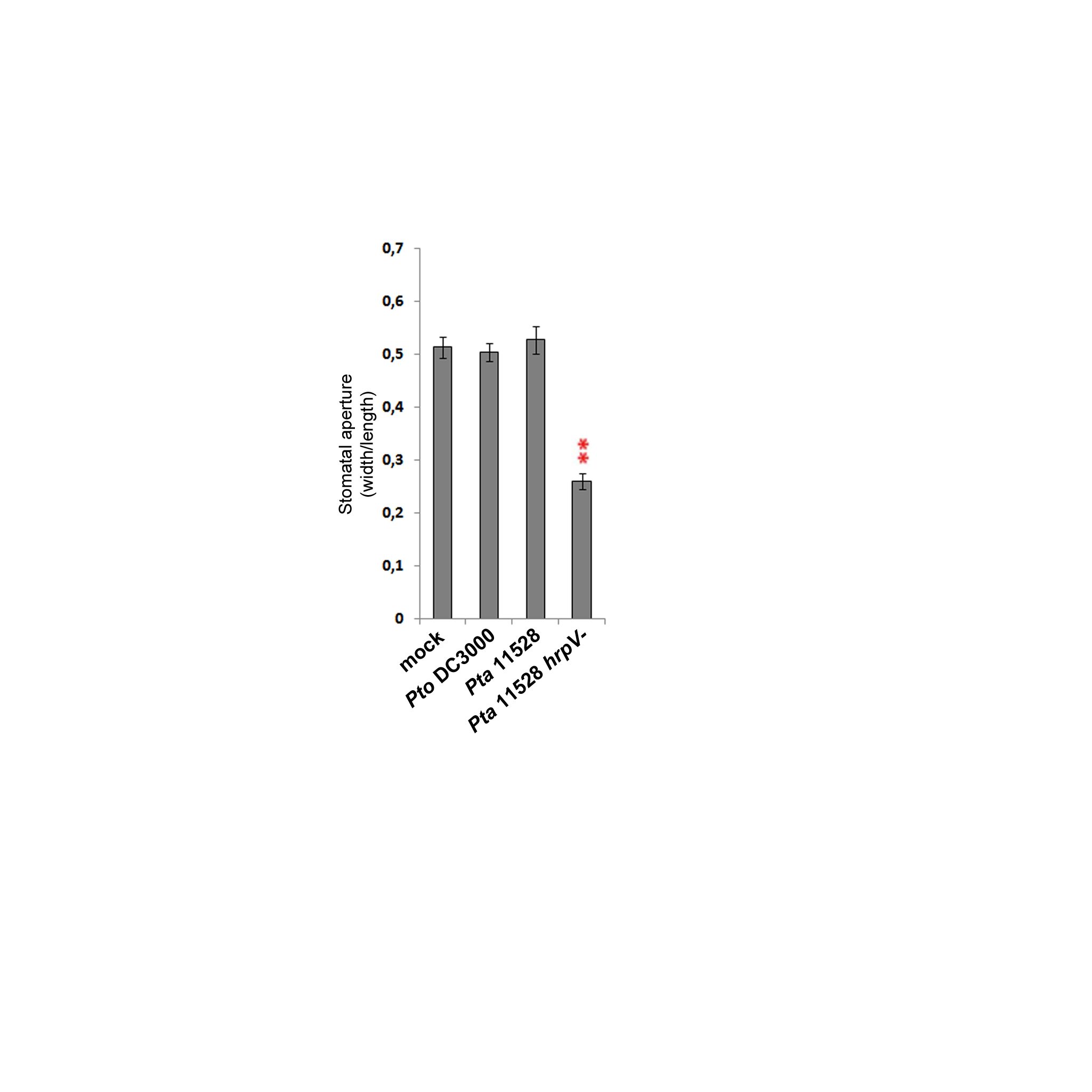

Supplement: Figure S1 — Live Pta 11528 bacteria manipulate stomatal aperture in a TTSS-dependent manner. Stomatal aperture in wild-type N. benthamiana leaves measured after 5 hours of incubation with mock or bacterial strains Pto DC3000, Pta 11528, or Pta 11528 hrpV−. Error bars indicate SEM (n = 17). Asterisks indicate significant differences compared with mock-treated samples at **p<0.01. The results are representative of three independent experiments. (TIF) [file pbio.1001792.s001.tif]

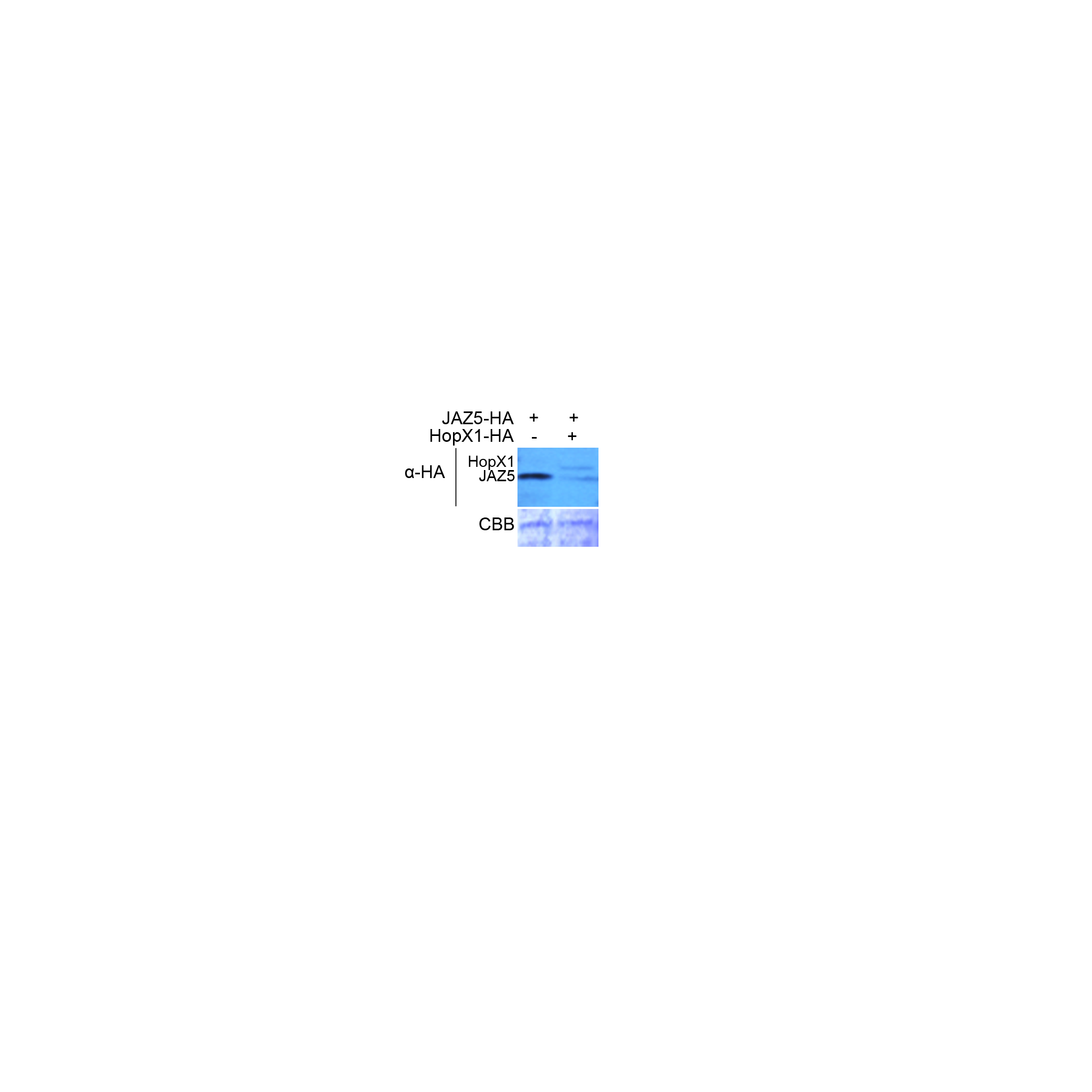

Supplement: Figure S2 — HopX1 compromises the accumulation of JAZ5. Immunoblots showing JAZ5-HA accumulation in the presence of HopX1-HA when co-expressed transiently in N. benthamiana for two days. Proteins were detected with anti-HA antisera. CBB, Coomassie brilliant blue staining. The results are representative of three independent experiments. (TIF) [file pbio.1001792.s002.tif]

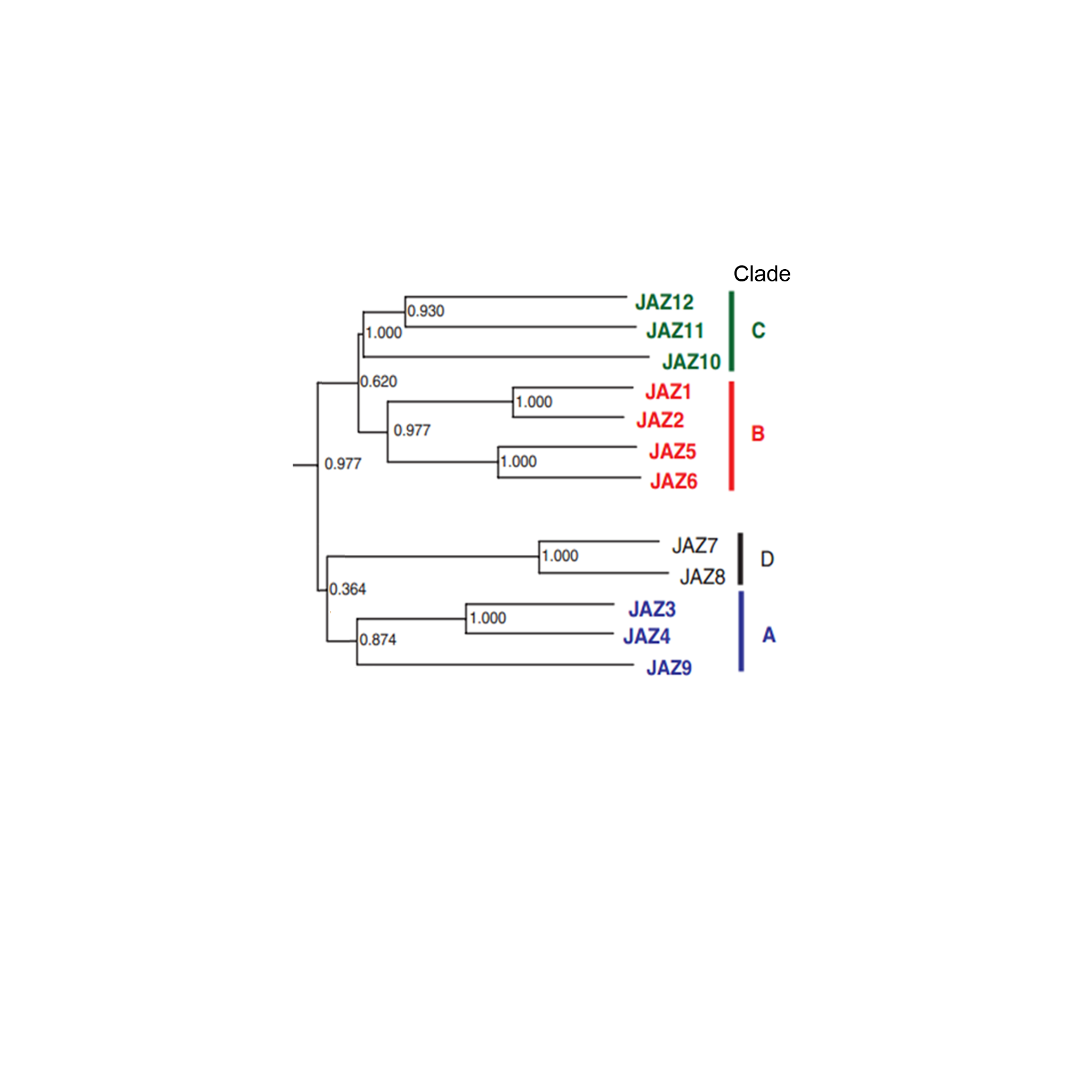

Supplement: Figure S3 — Phylogenetic tree of the Arabidopsis JAZ proteins. Phenogram representation of the neighbor-joining phylogenetic tree of the 12 full-length JAZ proteins. The sequence alignment was generated using DiAlign (Genomatix) and the tree was created by Phylodendron (University of Indiana). Branch lengths are proportional to the estimated evolutionary distance. Bootstrap values are included. JAZ proteins can be tentatively grouped into four clades. (TIF) [file pbio.1001792.s003.tif]

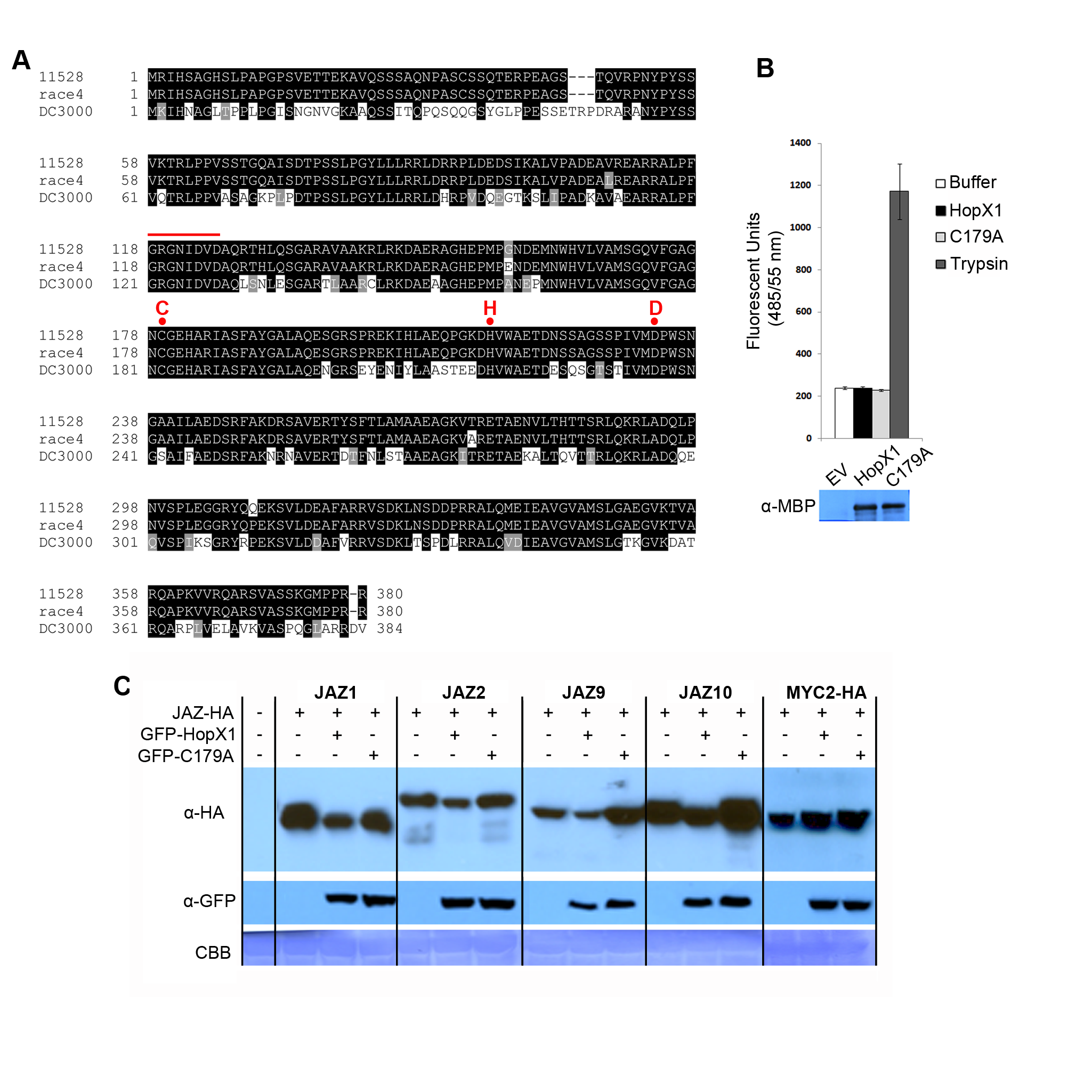

Supplement: Figure S4 — HopX family members contain a consensus cysteine-based catalytic triad and a conserved N-terminal domain. (A) HopX family members contain a consensus cysteine-based catalytic triad and a conserved N-terminal domain. Conservation of the putative catalytic residues (red dots) and the N-terminal domain (red line) between P. syringae HopX1 alleles from Pta 11528, Pto DC3000, and Pph race 4. Sequences were downloaded from the National Center for Biotechnology Information and were aligned using DiAlign (Genomatix). (B) MBP-HopX1 has no protease activity in vitro on the general substrate casein. Protease activity of recombinant MBP-HopX1 or MBP-HopX1C179A incubated with fluorescein isothiocyanate (FITC)-labeled casein at 37°C overnight with moderate shaking. Trypsin was used as a positive control. Fluorescence units record the fluorescence intensity with excitation at 485 nm and monitor the emission wavelength of 535 nm (485/535 nm). An immunoblot showing MBP-HopX1 and MBP-HopX1C179A-HA effector inputs is also shown. This experiment was repeated three times with similar results. (C) Degradation of JAZ1, JAZ2, JAZ9 and JAZ10 by HopX1 requires the cysteine-based catalytic triad of a putative protease in vivo. The immunoblots show JAZ1-HA, JAZ2-HA, JAZ9-HA, JAZ10-HA and MYC2-HA accumulation in the presence of GFP-HopX1 or GFP-HopX1C179A when co-expressed transiently in N. benthamiana. This experiment was repeated twice with similar results. (TIF) [file pbio.1001792.s004.tif]

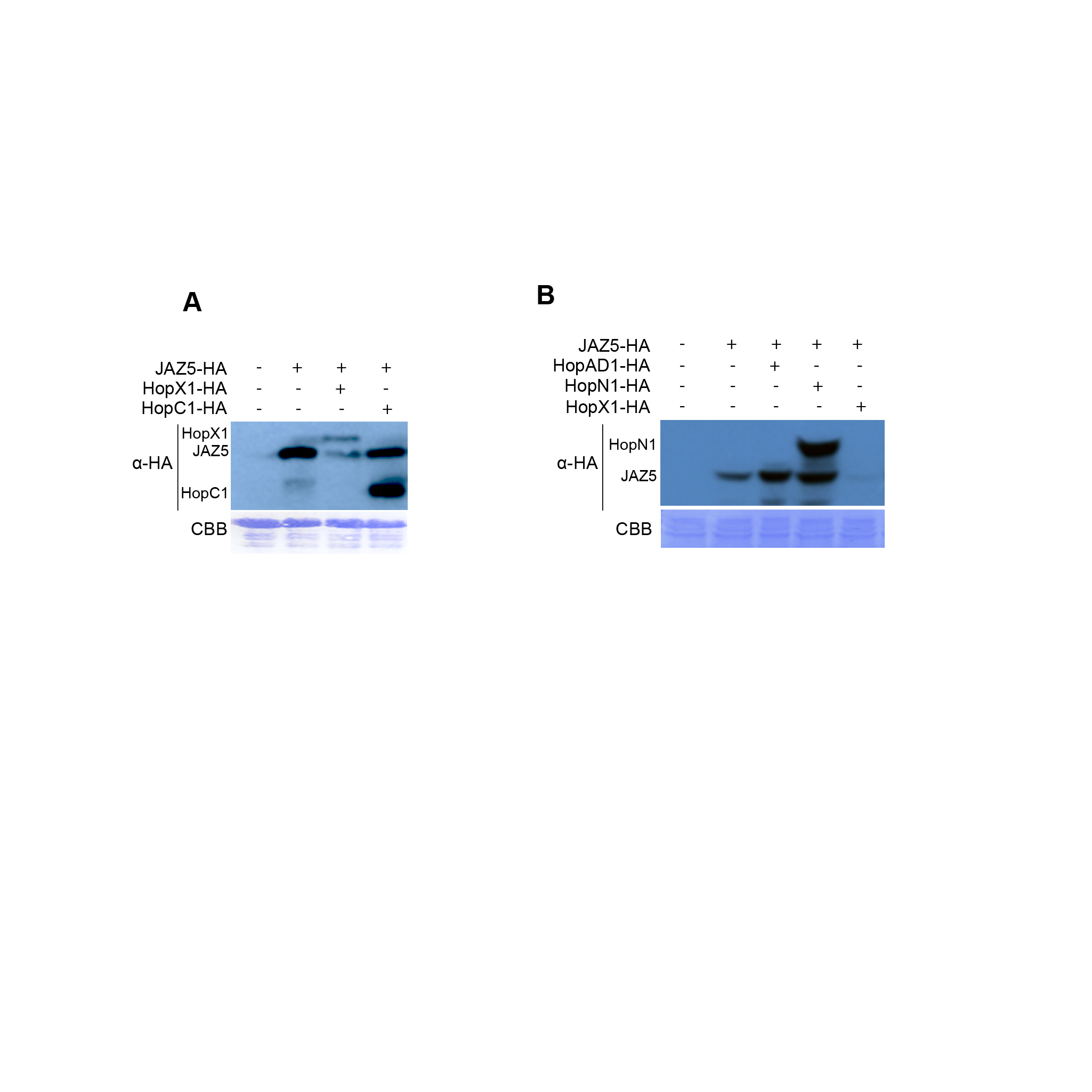

Supplement: Figure S5 — The cysteine proteases HopC1 and HopN1, or the unrelated effector HopAD1-HA, do not compromise JAZ accumulation. (A) Immunoblots showing JAZ5-HA accumulation in the presence of HopX1-HA, the cysteine protease HopC1-HA or an empty vector control when co-expressed transiently in N. benthamiana for 2 days. Proteins were detected with anti-HA antisera. CBB, Coomassie brilliant blue staining. This experiment was repeated twice with similar results. (B) Immunoblots showing JAZ5-HA accumulation in the presence of HopX1-HA, the cysteine protease HopN1-HA, the unrelated effector HopAD1-HA, or an empty vector control when co-expressed transiently in N. benthamiana for 2 days. Proteins were detected with anti-HA antisera. CBB is shown. This experiment was repeated three times with similar results. (TIF) [file pbio.1001792.s005.tif]

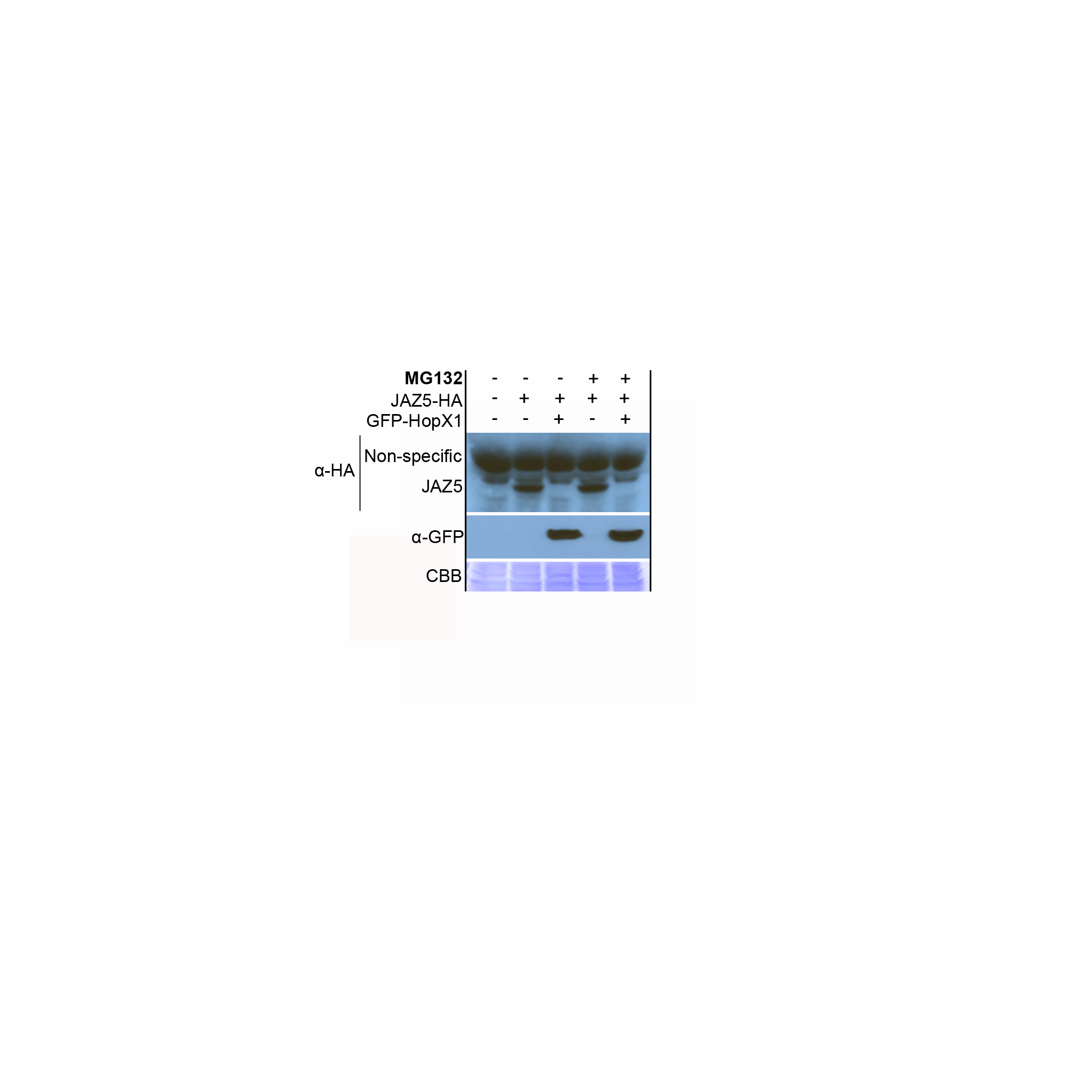

Supplement: Figure S6 — JAZ5 degradation by HopX1 is independent of the 26S proteasome. Immunoblots showing JAZ5-HA accumulation in the presence of GFP-HopX1 or an empty vector control when co-expressed transiently in N. benthamiana for 2 days after treatment with 100 µM of MG132. This experiment was repeated twice with similar results. (TIF) [file pbio.1001792.s006.tif]

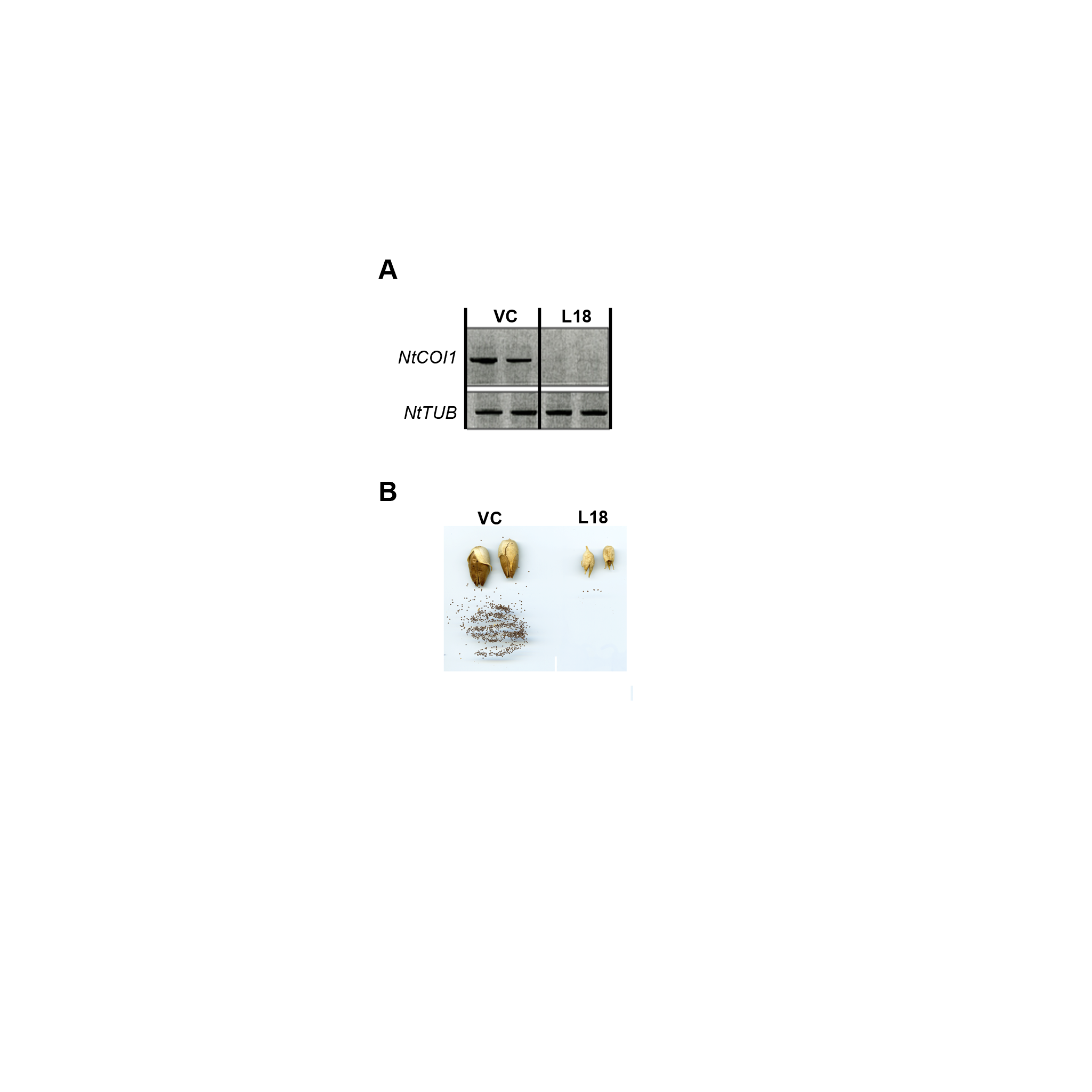

Supplement: Figure S7 — Molecular and phenotypic analysis of NtCOI1 -silenced N. tabacum plants. (A) RT-PCR of NtCOI1 expression in leaves of N. tabacum-silenced EV plants (Line VC) or N. tabacum plants silenced for the NtCOI1 gene (Line L18). N. tabacum α-Tubulin (NtTUB) was used as an amplification control. This supporting figure is related to Figure 3A. (B) Capsule and seeds production obtained from transgenic N. tabacum plants silenced with an EV construct (Line VC) or NtCOI1 (Line 18). (TIF) [file pbio.1001792.s007.tif]

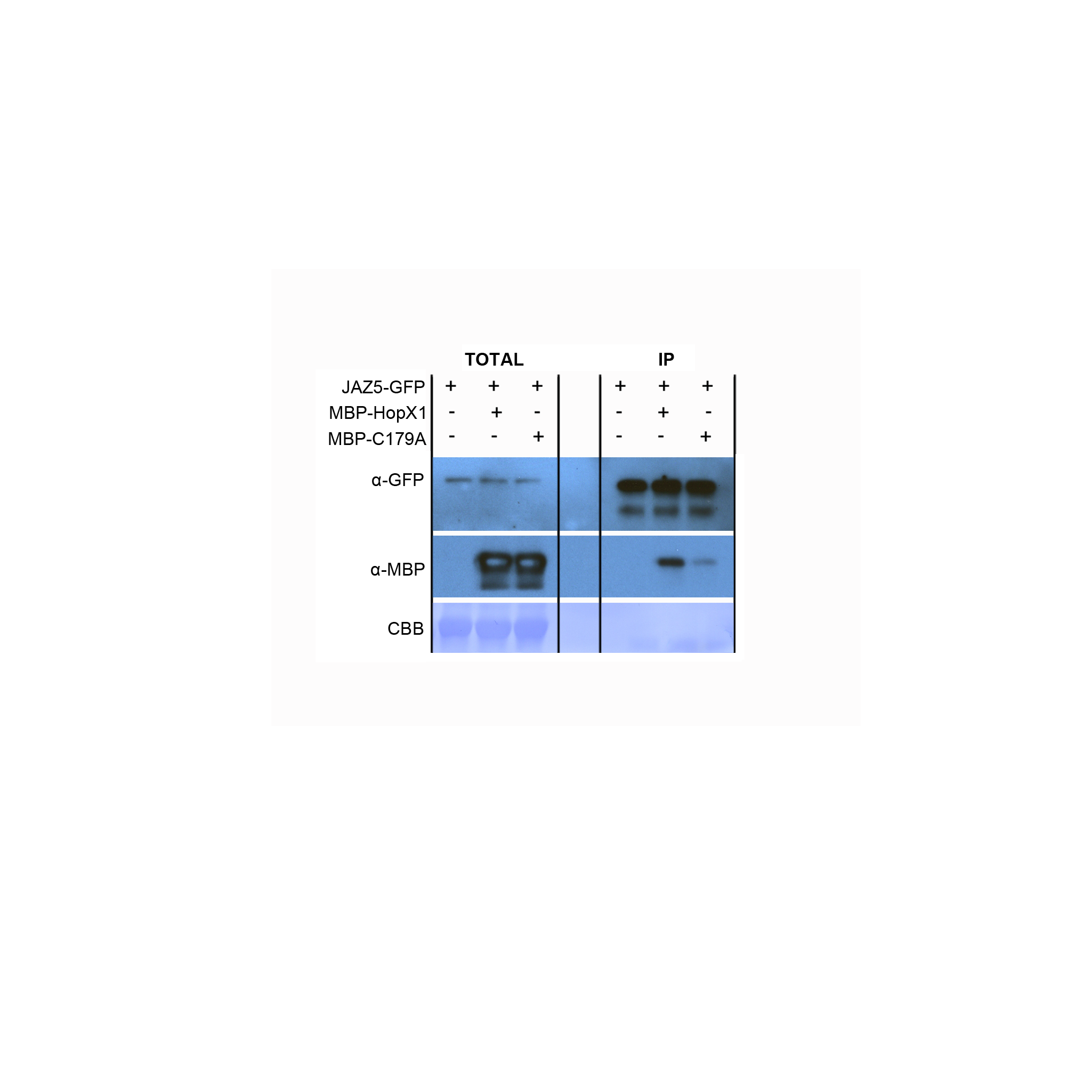

Supplement: Figure S8 — MBP-HopX1 co-immunoprecipitates with JAZ5-GFP from N. benthamiana plant extracts. MBP-HopX1 and MBP- HopX1C179A proteins purified from E. coli cells were incubated 2 hours with N. benthamiana plants extracts transiently expressing the JAZ5-GFP transgene and then subjected to co-immunoprecipitation analysis using GFP agarose beads. CBB, Coomassie brilliant blue staining. This experiment was repeated twice with similar results. (TIF) [file pbio.1001792.s008.tif]

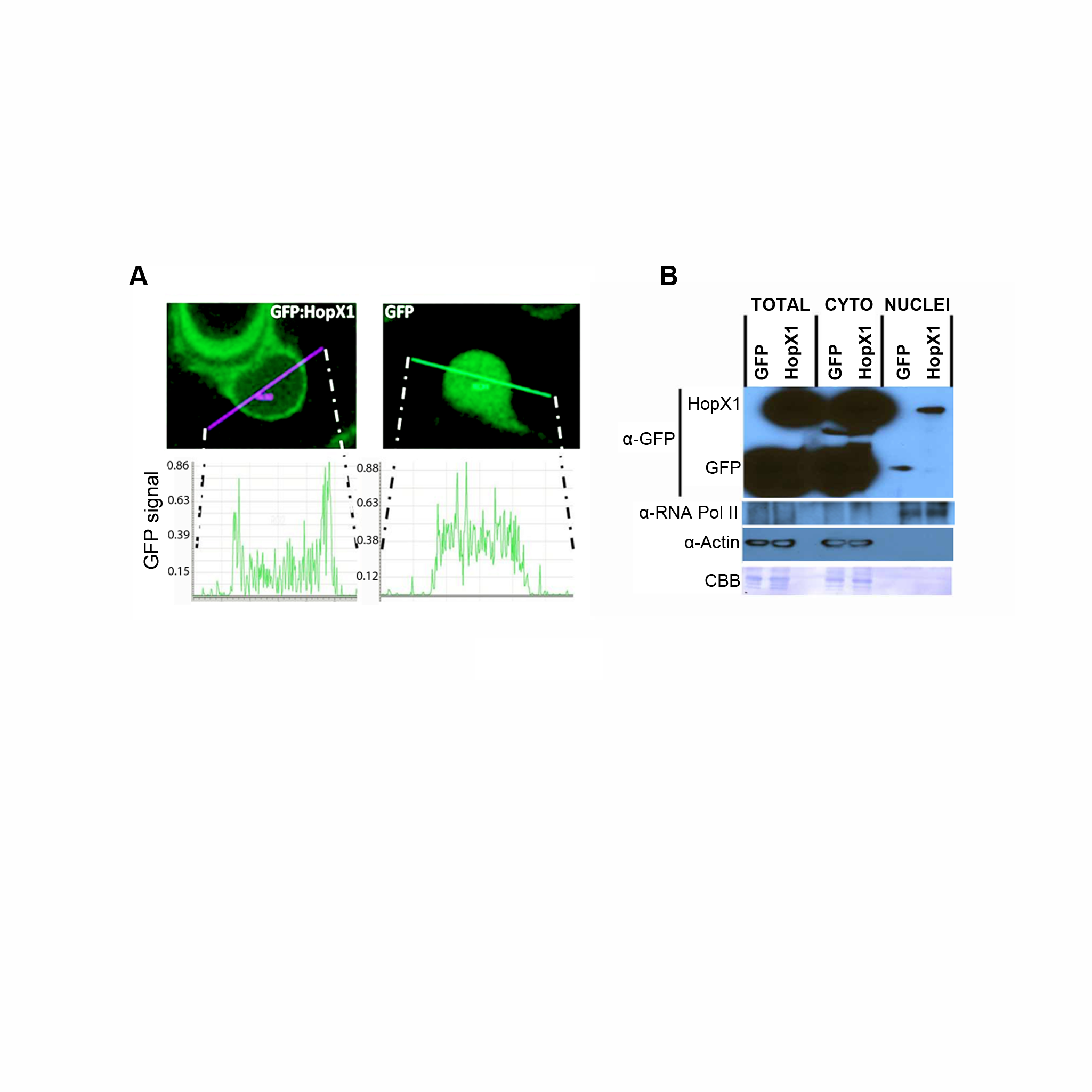

Supplement: Figure S9 — HopX1 subcellular localization in plant cells. (A) Confocal microscopy localization of GFP- HopX1 or GFP alone in transiently transformed N. benthamiana leaves 48 hours post-infiltration (green). The nuclear GFP signal of a middle cross section is shown. This supporting figure is related to Figure 3F. Note that the pictures show confocal sections of the nuclei and, therefore, the signal is nuclear and not derived from GFP protein surrounding the nuclear membrane. (B) Crude subcellular fractionation of GFP-HopX1 in N. benthamiana. Immunoblots showing GFP-HopX1 localization after subcellular fractionation when GFP-HopX1 or GFP alone was expressed transiently in N. benthamiana leaves for 48 hours. Total, cytoplasmic and nuclear fractions are shown. Actin, cytoplasmic intrinsic protein for control of the purity of the nuclear fraction. RNA polymerase II, nuclear intrinsic protein for control of enrichment of the nuclear fraction. Equivalent fraction volumes were loaded. This experiment was repeated twice with similar results. (TIF) [file pbio.1001792.s009.tif]

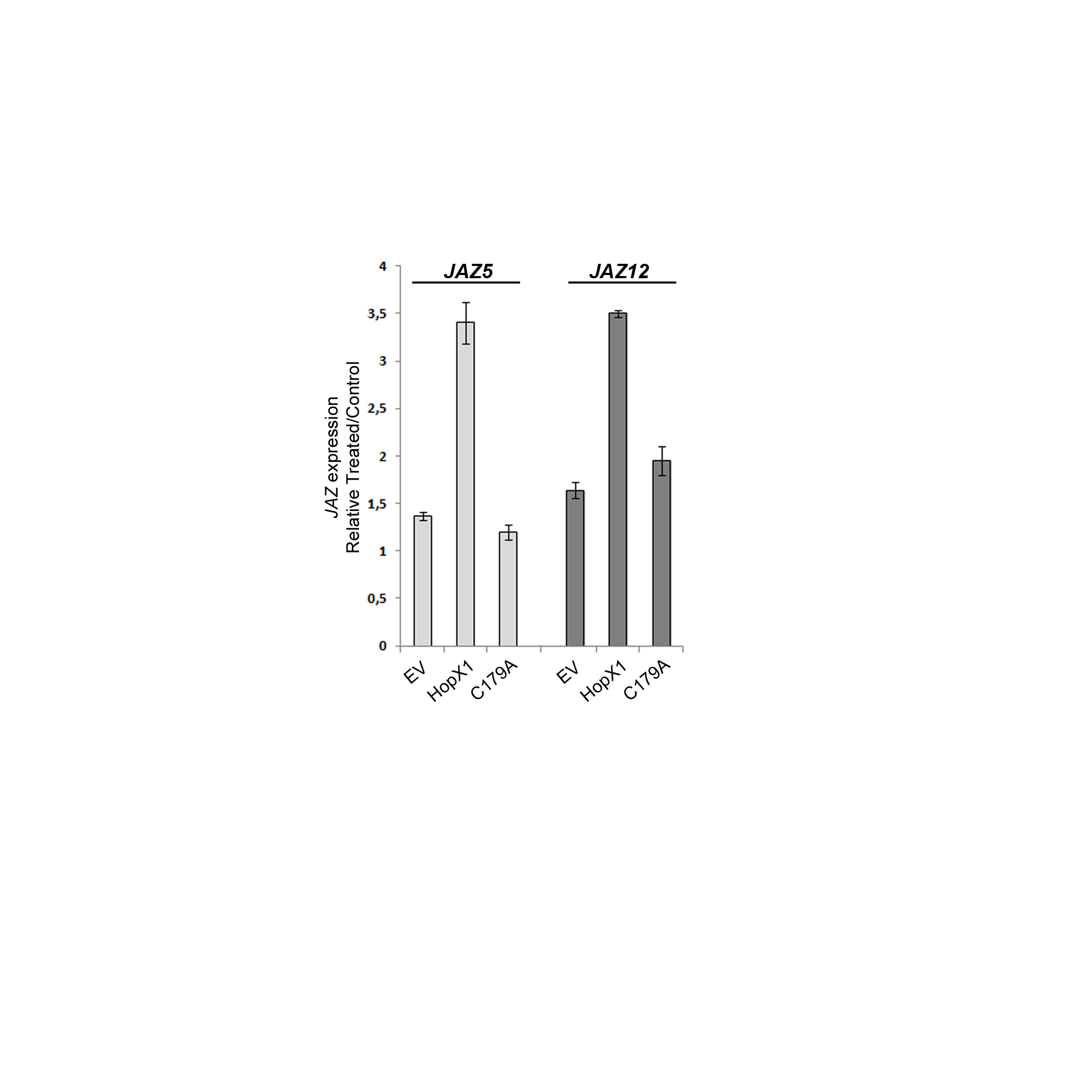

Supplement: Figure S10 — HopX1 triggers the activation of JA-dependent gene expression in Arabidopsis . Quantitative RT-PCR analysis of JAZ5 and JAZ12 expression on Col-0 (EV) and stable transgenic Arabidopsis Aa–0 lines expressing the hopX1 or hopX1 C179A genes 36 hours after treatment with DEX or a mock solution. The measurements (three technical replicates) represent the relative expression levels between mock (control) and DEX-treated plants in each Arabidopsis background. All samples were normalized against the housekeeping gene AtACT8. Error bars represent standard deviation (SD). The results are representative of three independent experiments. (TIF) [file pbio.1001792.s010.tif]

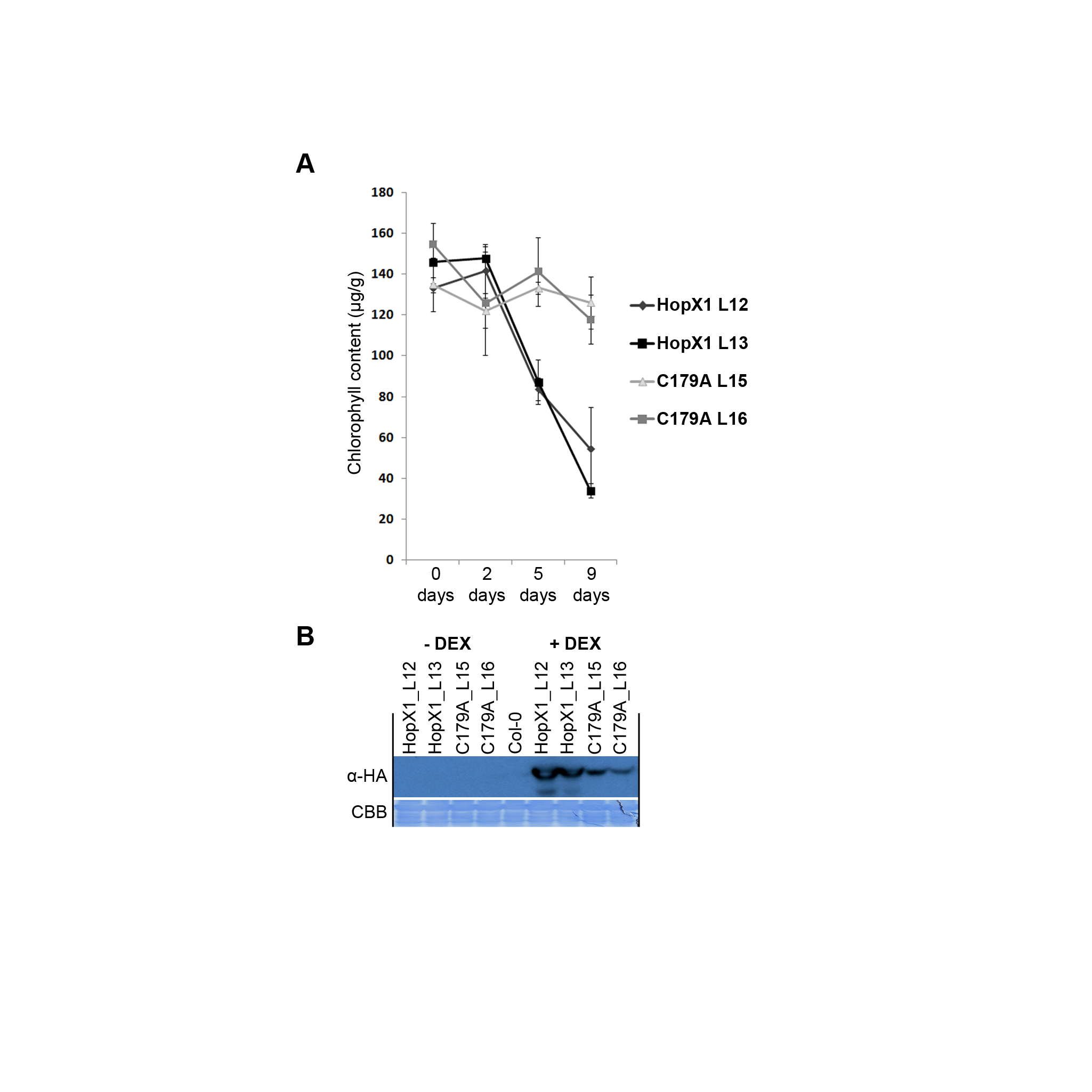

Supplement: Figure S11 — Ectopic expression of hopX1 but not hopX1 C179A in stable transgenic Arabidopsis Aa–0 correlates with loss in chlorophyll content. (A) Contents of leaf chlorophyll in stable transgenic Arabidopsis lines expressing the hopX1 or hopX1 C179A genes two, five, and nine days after DEX treatment. Error bars represent standard deviation (SD). L12 and L13 are two independent stable transgenic Arabidopsis lines expressing the hopX1 gene. L15 and L16 are two independent stable transgenic Arabidopsis lines expressing the hopX1 C179A gene. The results are representative of three independent experiments. (B) Immunoblots showing HopX1-HA or HopX1C179A-HA accumulation in stable Arabidopsis lines expressing the hopX1 or hopX1 C179A genes. Plants were induced with DEX or a mock solution for five hours. Line designations are as for Figure S11A. (TIF) [file pbio.1001792.s011.tif]

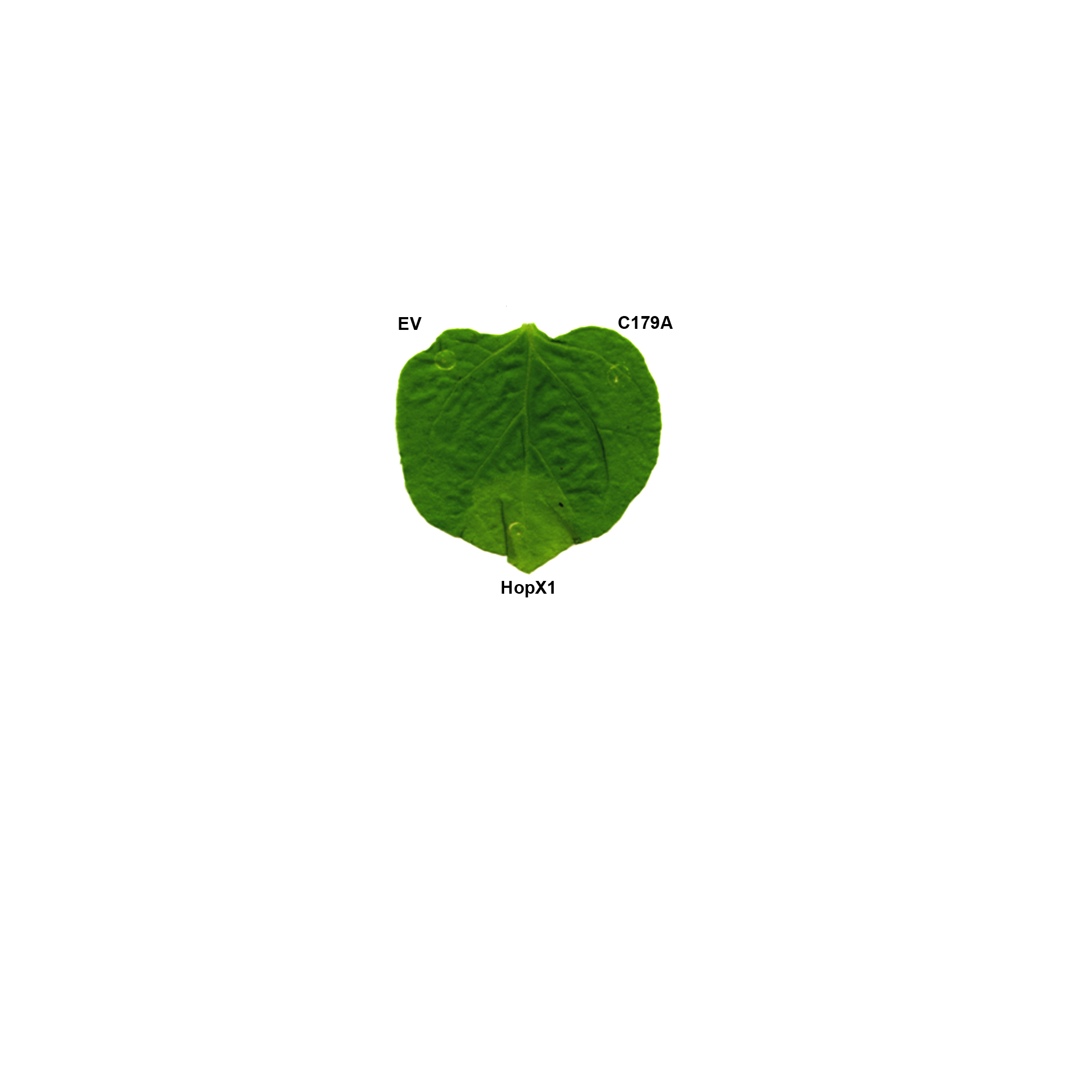

Supplement: Figure S12 — HopX1 induces chlorosis in N. benthamiana leaves when transiently expressed. Chlorotic symptoms in transiently expressed EV, hopX1, or hopX1 C179A N. benthamiana leaf tissue after DEX treatment for 48 hours. (TIF) [file pbio.1001792.s012.tif]

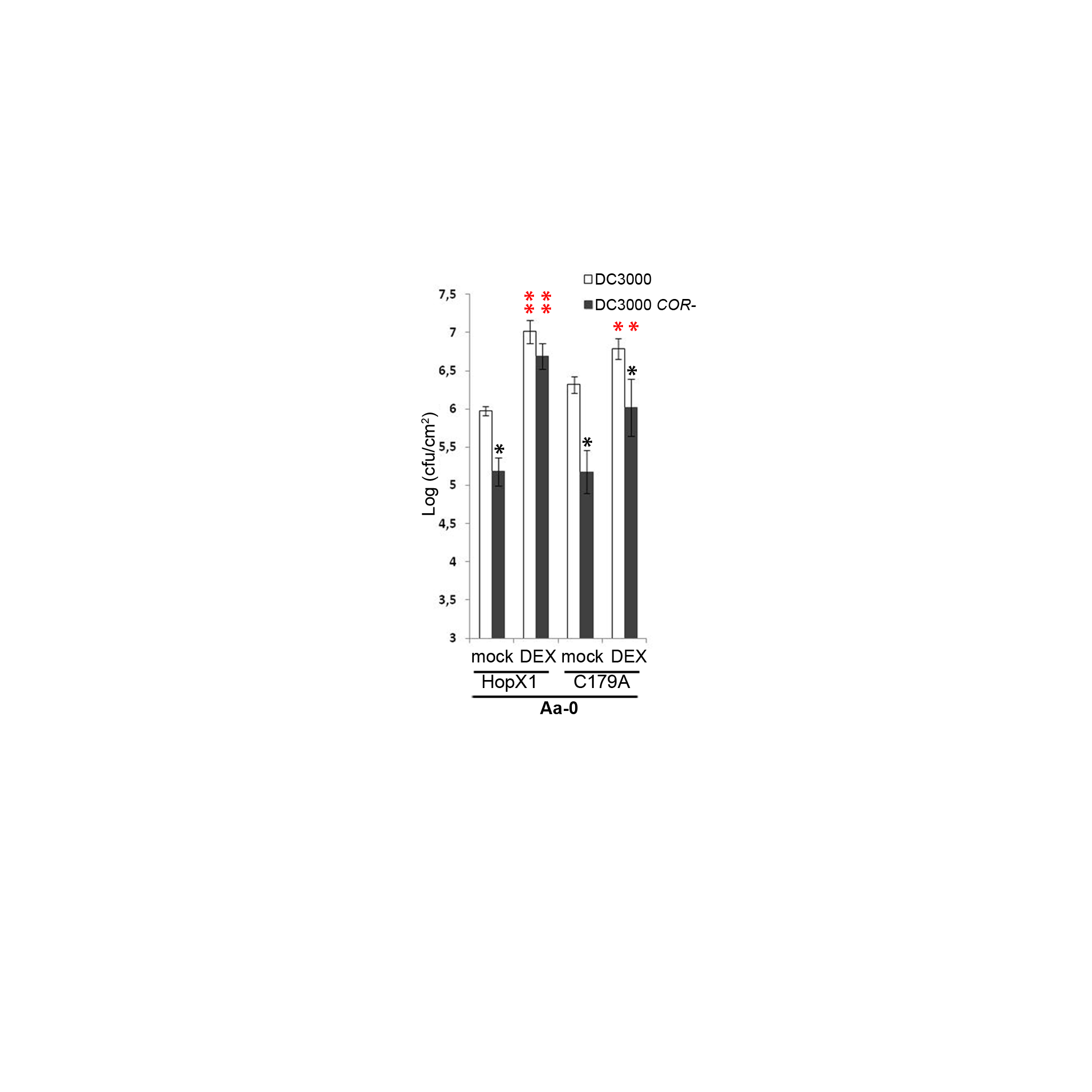

Supplement: Figure S13 — HopX1 complements the growth of a COR-deficient Pto DC3000 strain when expressed ectopically in Arabidopsis Aa–0 plants. Growth of Pto DC3000 and the isogenic Pto DC3000 COR− strain on stable transgenic Arabidopsis lines expressing the hopX1 or hopX1 C179A genes two days after spray inoculation with bacteria at 108 cfu/ml−1. Plants were pre-treated with DEX or a mock solution 24 hours prior to bacterial infection. Pto DC3000 growth increased by about one log (cfu/cm2) in transgenic HopX1 Arabidopsis pre-treated with DEX compared to mock treated control plants whereas ectopic expression of hopX1 C179A could only promote Pto DC3000 growth to about half a log (cfu/cm2). The data indicate that HopX1 contributes to pathogenicity over 2-fold compared to HopX1C179A when effectors are ectopically overexpressed in Arabidopsis plants. Furthermore, in mock treated transgenic HopX1 Arabidopsis plants, Pto DC3000 COR− growth was restricted by one log (cfu/cm2) compared to Pto DC3000. However, both strains grew to similar levels when expressing hopX1 in response to DEX treatment. In contrast, Pto DC3000 COR− growth returned to wild-type levels when HopX1 carried the C179A mutation. Error bars indicate standard error of the mean (SEM). Red asterisks indicate statistically significant differences between DEX and mock-treated plants in each Arabidopsis line/bacterial strain (Student's t test, *p<0.05 and **p<0.01). Black asterisks indicate statistically significant differences between Pto DC3000 and Pto DC3000 COR− in each condition (Student's t test, *p<0.01). The results are representative of three independent experiments. (TIF) [file pbio.1001792.s013.tif]

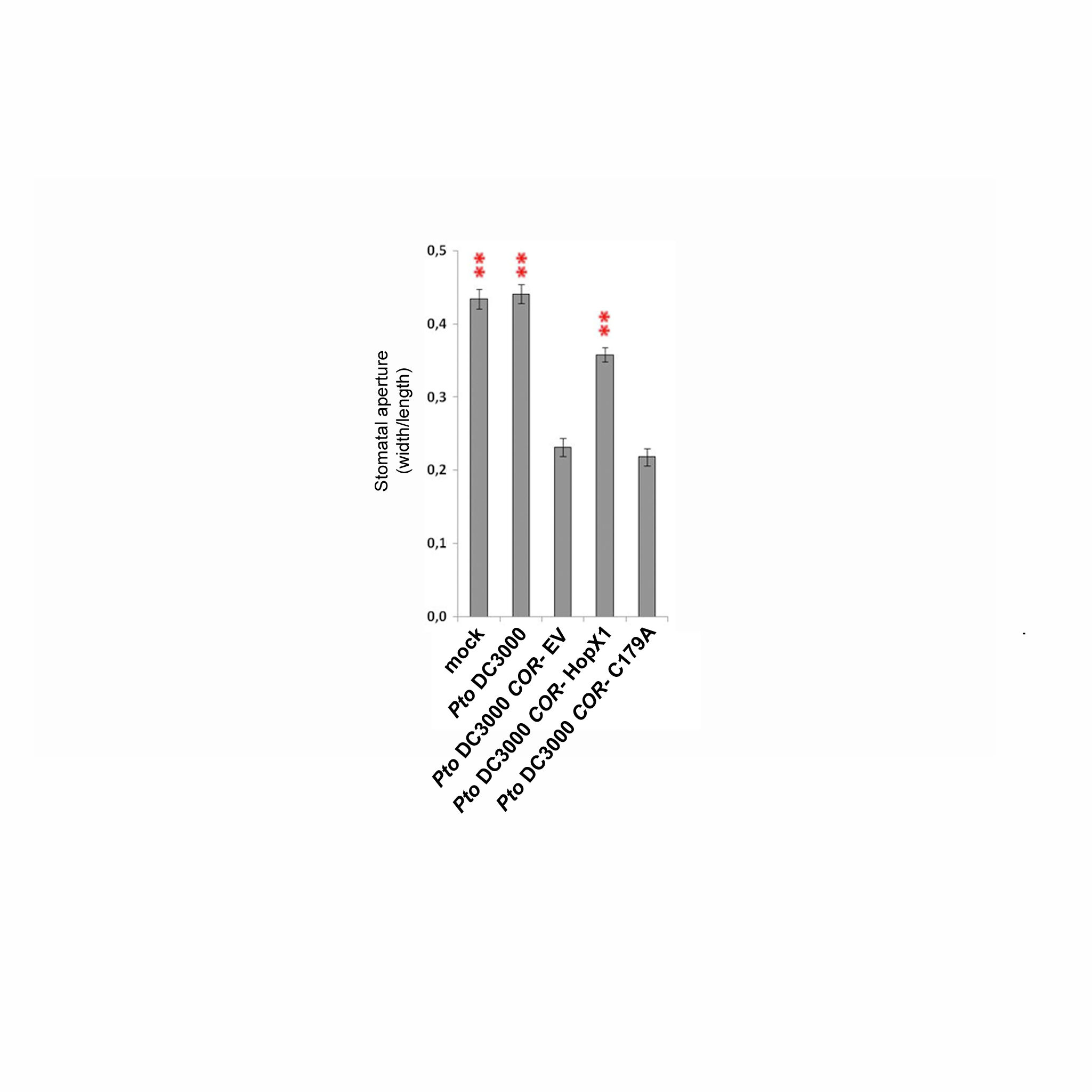

Supplement: Figure S14 — Pto DC3000 COR− bacteria expressing hopX1 manipulate stomatal aperture. Stomatal aperture in wild-type N. benthamiana leaves measured after 5 hours of incubation with mock, Pto DC3000, or Pto DC3000 COR− bacteria expressing hopX1, hopX1 C179A, or an EV control. Error bars indicate SEM (n = 20). Asterisks indicate significant differences compared with Pto DC3000 COR− bacteria expressing an EV control at **p<0.01. The results are representative of two independent experiments. (TIF) [file pbio.1001792.s014.tif]

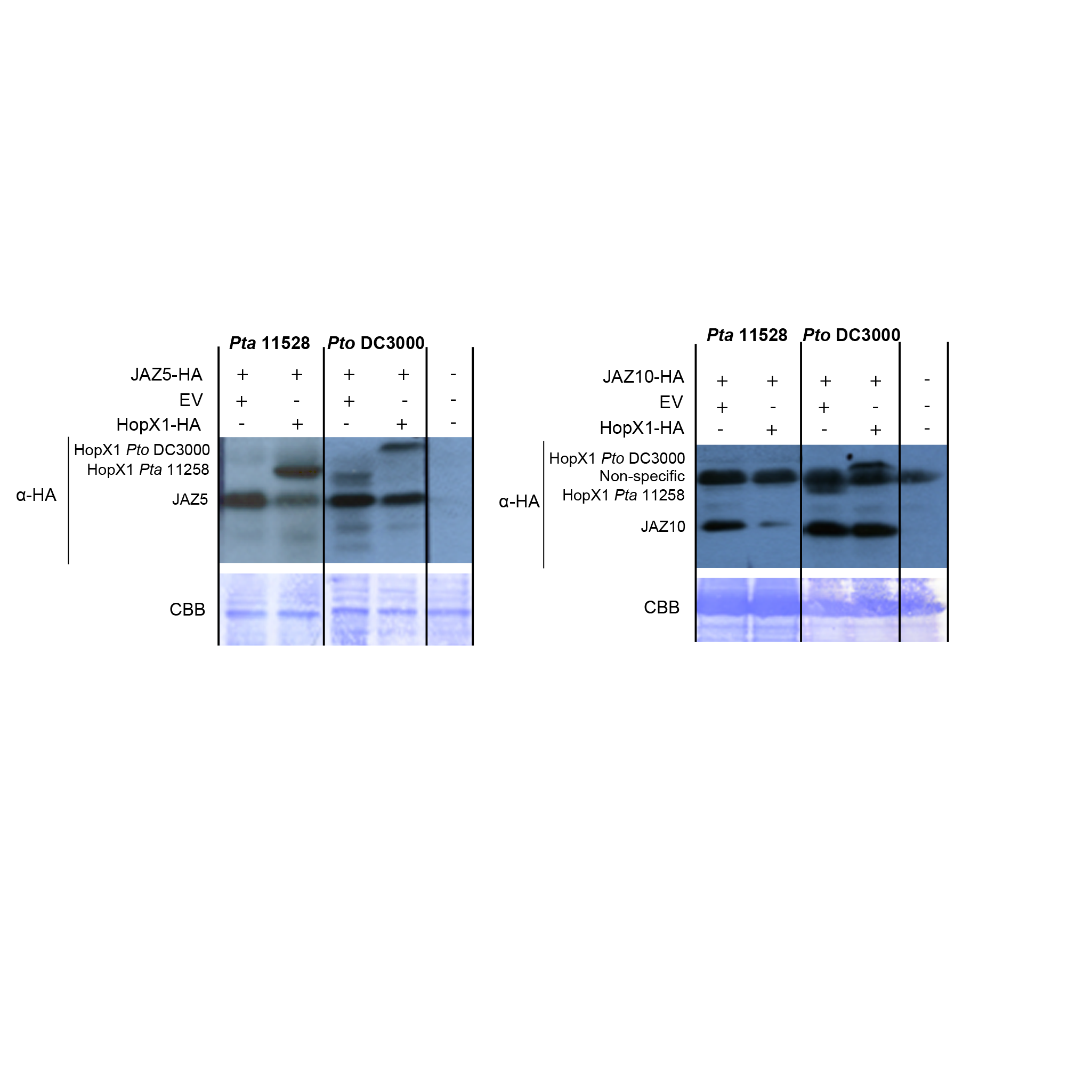

Supplement: Figure S15 — HopX1 Pto DC3000 does not compromise the accumulation of JAZs when transiently co-expressed in N. benthamiana . Immunoblots showing JAZ5-HA and JAZ10-HA accumulation in the presence of HopX1Pta 11528, HopX1Pto DC3000, or an empty vector control when co-expressed transiently in N. benthamiana. Proteins were detected with anti-HA. CBB, Coomassie brilliant blue staining. A non-specific band is shown as an additional loading control in the blot of JAZ10. This experiment was repeated twice with similar results. (TIF) [file pbio.1001792.s015.tif]

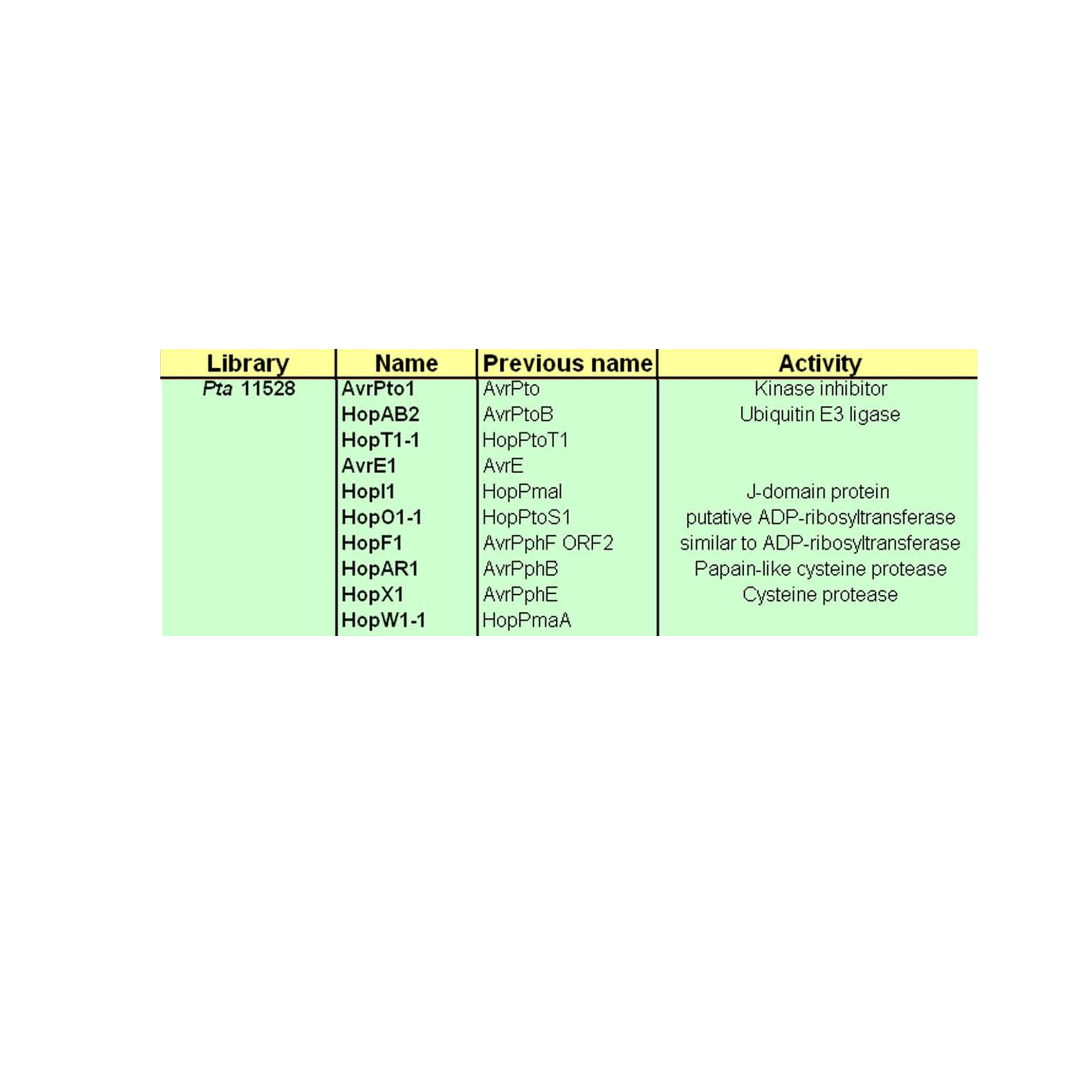

Supplement: Table S1 — List of Pta 11528 secreted effector proteins included in this study. Putative activities of type III effectors are shown. The Pta 11528 effector identity was determined by BLAST against all known effector proteins. (TIF) [file pbio.1001792.s016.tif]
